# Rap1-GTPases control mTORC1 activity by coordinating lysosome organization with amino acid availability

**DOI:** 10.1038/s41467-020-15156-5

**Published:** 2020-03-17

**Authors:** Anders P. Mutvei, Michal J. Nagiec, Jens C. Hamann, Sang Gyun Kim, C. Theresa Vincent, John Blenis

**Affiliations:** 1 Weill Cornell Medicine, Sandra and Edward Meyer Cancer Center, Belfer Research Building, 413 E. 69th St., New York, NY 10021 USA; 2 Karolinska Institutet, Department of Microbiology, Tumor and Cell biology, Nobels väg 16, KI Solna Campus Karolinska Institutet, Box 280, SE-171 77 Stockholm, Sweden; 3 Department of Immunology, Genetics and Pathology, Uppsala University, Rudbeck Laboratory, 751 85 Uppsala, Sweden; 4 Department of Microbiology, New York University School of Medicine, New York, NY USA

**Keywords:** Cell biology, Cell signalling, Molecular biology

## Abstract

The kinase mTOR complex 1 (mTORC1) promotes cellular growth and is frequently dysregulated in cancers. In response to nutrients, mTORC1 is activated on lysosomes by Rag and Rheb guanosine triphosphatases (GTPases) and drives biosynthetic processes. How limitations in nutrients suppress mTORC1 activity remains poorly understood. We find that when amino acids are limited, the Rap1-GTPases confine lysosomes to the perinuclear region and reduce lysosome abundance, which suppresses mTORC1 signaling. Rap1 activation, which is independent of known amino acid signaling factors, limits the lysosomal surface available for mTORC1 activation. Conversely, Rap1 depletion expands the lysosome population, which markedly increases association between mTORC1 and its lysosome-borne activators, leading to mTORC1 hyperactivity. Taken together, we establish Rap1 as a critical coordinator of the lysosomal system, and propose that aberrant changes in lysosomal surface availability can impact mTORC1 signaling output.

## Abstract (web summary)

mTORC1 regulates cellular growth from the lysosome, but how changes in the organisation of the lysosomal system affect mTORC1 signalling is poorly understood. Here the authors show that amino acid-dependent Rap1-mediated changes in lysosomal abundance determine overall signalling output of mTORC1.

## Introduction

The mTOR complex 1 (mTORC1) protein kinase integrates nutrient signals to drive cellular growth and is dysregulated in multiple diseases, including cancer^1,2^. When cells are supplied with nutrients, mTORC1 is recruited to lysosomes and late endosomes (subsequently referred to as lysosomes) where it is activated and promotes various biosynthetic processes, including protein, nucleotide and lipid synthesis^3^. mTORC1 activation is mediated by Rag-GTPases, which recruit and anchor mTORC1 to the lysosomal surface where the Rheb-GTPase stimulates its phosphotransferase activity^3–5^. Despite important advances in this field^1–4^, how mTORC1 signaling is suppressed when nutrients are limited remains incompletely understood. Here, we report that when amino acids are limited, the Rap1-GTPases concentrate lysosomes to the perinuclear region and reduce overall lysosome abundance, which suppresses mTORC1 signaling. This is due to a Rap1-mediated decrease in the surface available for mTORC1 activation. In contrast, Rap1 depletion leads to an expansion of the lysosome system and mTORC1 hyperactivation, as a result of the increased association between mTORC1 and its lysosome-bound activators. We propose that aberrant changes in lysosome abundance impact overall mTORC1 signaling output.

## Results

### Rap1 suppresses mTORC1 signaling

**Fig. 1:**
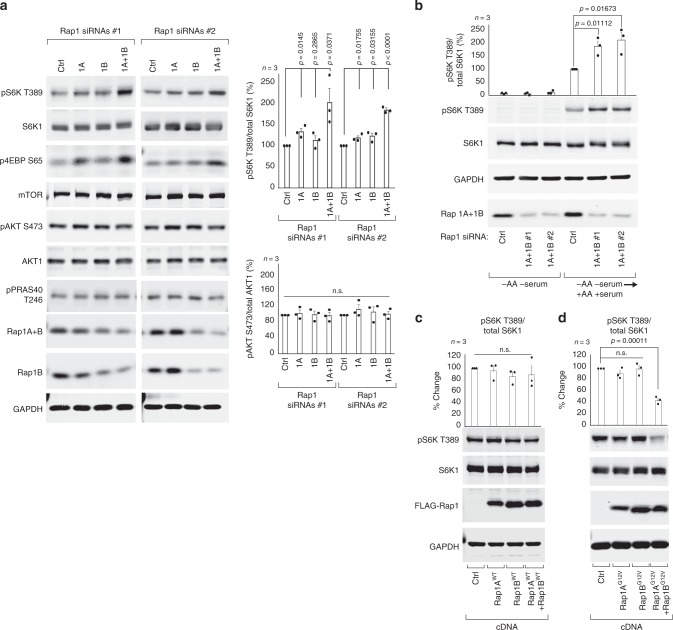
Rap1 suppresses basal mTORC1 signaling. In **a**, **b** mTORC1 associated signaling was assessed in HEK293A cells after siRNA depletion of Rap1A, Rap1B or both Rap1A and Rap1B, by immunoblotting endogenous proteins, as indicated. Quantifications are shown to the right and in Supplementary Fig. 1. In the bottom graph, *p* = 0.888 and *p* = 0.724 for pAKT S473 siRNA Ctrl vs Rap1A+B#1 and Ctrl vs. Rap1A+B #2, respectively. In **b** cells were depleted of serum and amino acids for 60 min and stimulated with full growth media for 10 min. In **c**, **d** HEK293T cells were transfected with an equal amount of the indicated cDNA vectors and serum deprived before subjected to immunoblotting. Graphs represent relative immunoblot band intensity from three individual experiments (*n* = 3). Statistical data are presented as mean values  ± s.e.m. n.s. = not significant (*P* > 0.05); Student’s *t*-test; two-sided, unpaired. pS6K T389 and total S6K1 were processed on separate blots due to technical reasons. In **c** *p* = 0.6730, 0.1417 and 0.1532 for EV vs Rap1AWT, Rap1BWT and Rap1A+BWT, respectively. In **d** *p* = 0.1798 and 0.9251 EV vs Rap1AG12V and Rap1BG12V, respectively. See Source Data File for statistics source data. Uncropped images of blots are shown in Supplementary Fig. 15.

To identify uncharacterized mTORC1 suppressors, we turned to our previous genome-wide small interfering RNA (siRNA) screen that was designed to identify novel mTORC1 regulators^6^. The evolutionarily conserved GTPase Rap1A was one of the top putative negative regulators identified in the screen and Rap1A is known to localize to endosomal membranes^7^, including the lysosome^8^ (Supplementary Fig. 1a, b) and the yeast vacuole^9^. Rap1A and its closely related isoform Rap1B are best known for their role in actin cytoskeleton organization, integrin activation and cell-cell contact formation and are not recognized as regulators of nutrient signaling^10^. To investigate if Rap1 depletion increases basal mTORC1 activity in full growth conditions, we targeted Rap1A, Rap1B or both isoforms in HEK293A cells, using two sets of siRNA (Supplementary Fig. 1c), and assessed the phosphorylation state of mTORC1 substrates T389 of S6K1 and S65 of eIF4E-binding protein 1 (4E-BP1). Depletion of both Rap1 isoforms resulted in mTORC1 hyperactivity, whereas depletion of either Rap1A or Rap1B alone gave rise to only a modest increase in mTORC1 signaling compared to control (Fig. 1a, Supplementary Fig. 1d), indicating that Rap1 suppresses basal mTORC1 signaling and that the two Rap1 isoforms exhibit cooperativity. mTORC1 hyperactivity after simultaneous Rap1A and Rap1B depletion was evident minutes after amino acid and serum stimulation (Fig. 1b), observed in multiple cell types (Supplementary Fig. 1f, g) and was not the result of increased growth factor signaling, as the phosphorylation status of AKT S473 (Fig. 1a, Supplementary Fig. 1f, g) or PRAS40 T246 (Fig. 1a, Supplementary Fig. 1e) remained unaffected in Rap1-depleted cells. The increase in basal mTORC1 activity was reversed by simultaneous expression of siRNA-resistant wild-type Rap1A and Rap1B (Supplementary Fig. 1h). To investigate if increased Rap1 activation limits mTORC1 signaling, we compared the effect of wild-type and constitutively active Rap1-G12V mutants on phosphorylation of downstream mTORC1 targets. Expression of individual wild-type or constitutively active G12V Rap1A and Rap1B isoforms did little to alter mTORC1 signaling (Fig. 1c, d), in line with previous observations^11,12^, whereas simultaneous expression of Rap1A-G12V and Rap1B-G12V (referred hereafter as Rap1-G12V) decreased basal mTORC1 signaling (Fig. 1d). Taken together, these results reveal a previously unknown role for Rap1 in suppressing mTORC1 signaling.

### Amino acid depletion induces Rap1 activity, independent of GCN2 and the Rag-GTPases

**Fig. 2:**
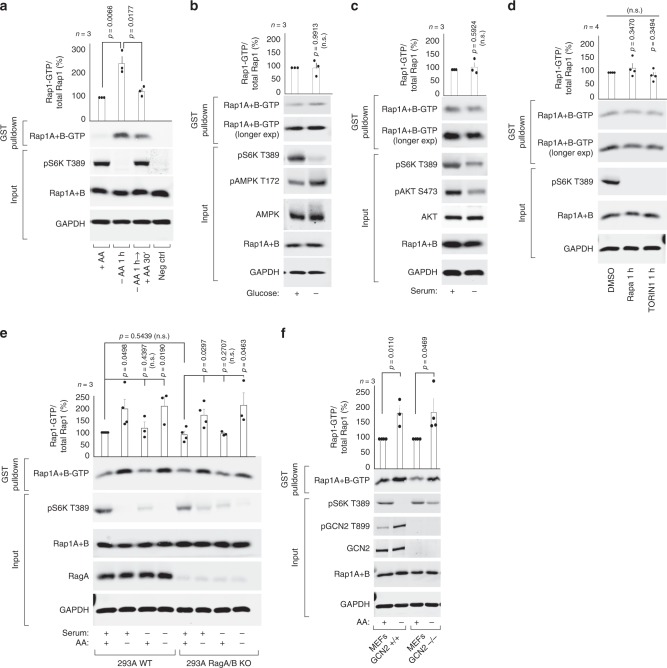
Amino acid deprivation induces Rap1 activity. In **a** through (**f**), endogenous Rap1 activity (Rap1A+B-GTP) was assessed through immunoprecipitation of GTP-bound Rap1 and immunoblotting against the indicated proteins. Cells had been subjected to 1 h depletion of all amino acids (−AA) (**a**, **e**, **f**), glucose (**b**) or serum (**c**, **e**), and re-stimulated with amino acids for 30 min (**a**), or treated for 1 h with 20 nM Rapamycin (Rapa), 50 nM Torin1 or DMSO control (**d**). Lysates were prepared from serum deprived HEK293T cells (**a**), HEK293A cells (**b**–**d**), RagA/B knockout HEK293A cells (**e**) or MEF cells deficient in GCN2 (**f**). Graphs represent relative immunoblot band intensity from *n* experiments: **a**, **b**, **c**, **e**, **f** *n* = 3, **d** *n* = 4. In **a**, **d**, **e**, **f** Rap1-GTP and total Rap1 were processed on separate blots due to technical reasons. Statistical data are presented as mean values ± s.e.m. n.s. = not significant (*P* > 0.05); Student’s *t*-test; two-sided, unpaired. See Source Data File for statistics source data. Uncropped images of blots are shown in Supplementary Fig. 16.

Basal mTORC1 signaling is suppressed in response to limitations in amino acids, energy sources or growth factors. To determine if Rap1 responds to changes in nutrient availability, we assessed endogenous Rap1-GTP loading in cells deprived of amino acids, glucose or serum, using a recombinant effector-binding assay that detects GTP-bound Rap1^13^. Strikingly, Rap1 activation was strongly increased upon acute amino acid starvation in multiple human cell types (Fig. 2a, Supplementary Fig. 1i, j), which was largely reversed by short-term amino acid replenishment (Fig. 2a). In contrast, endogenous Rap1-GTP loading was not increased by glucose or serum starvation (Fig. 2b, c). Importantly, pharmacological inhibition of mTORC1 using rapamycin or Torin1 demonstrated that Rap1 activation was not a consequence of mTORC1 inactivation (Fig. 2d). We next considered if Rap1 is regulated by known amino acid-responsive factors: the Rag-GTPase or the General Control Nonderepressible 2 (GCN2) signaling pathways^14^. Rap1 response to amino acid withdrawal was independent of both signaling pathways, as Rap1-GTP loading was readily increased in cells deficient for RagA and RagB (Fig. 2e), p14/LAMTOR2; a critical component of the guanine exchange factor complex for RagA/B^15^ (Supplementary Fig. 1k) or GCN2 (Fig. 2f). Together, these data suggest that amino acid starvation induces Rap1 activation independently of Rag-GTPase and GCN2 amino acid signaling pathways.

### Rap1 suppresses lysosome abundance and peripheral distribution when amino acids are limited

**Fig. 3:**
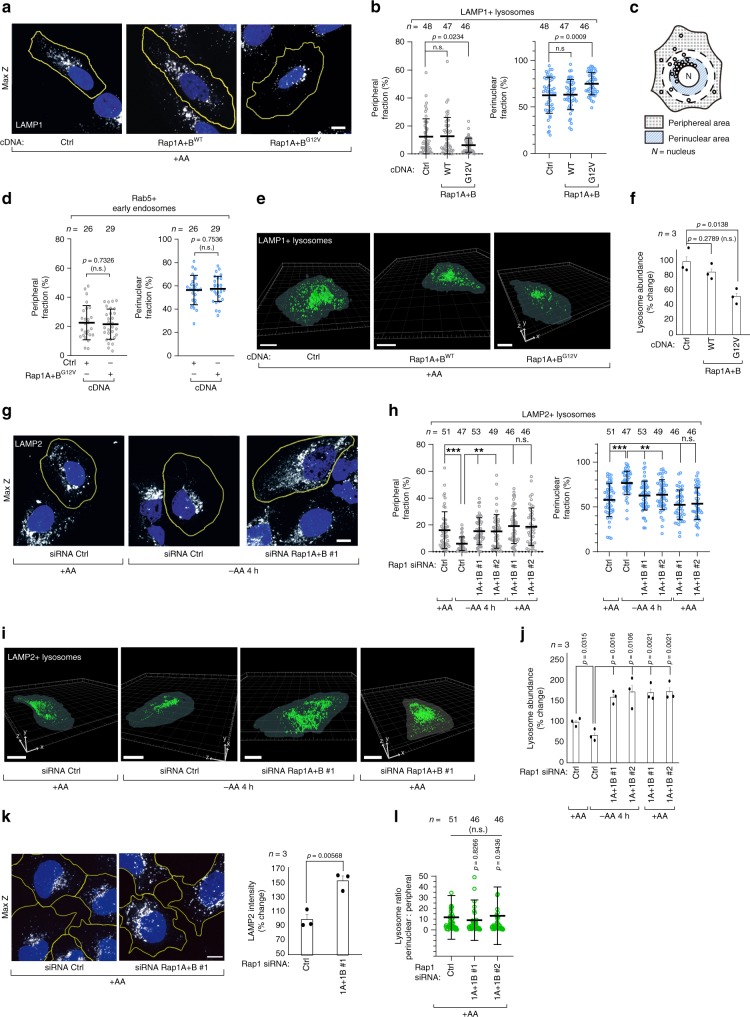
Rap1 suppresses lysosome abundance and peripheral distribution upon limitation in amino acids. **a**–**j** Effect of Rap1 activity on lysosome distribution and abundance. In **a**, **g** representative z-stack projections of endogenous lysosome distribution are shown for U2OS cells expressing wild-type (WT) or G12V Rap1A+B cDNA (**a**), or after 4 h of amino acid depletion (−AA 4 h) in combination with Rap1A+B depletion (**g**). The fraction of endosomes in the peripheral and perinuclear region was quantified in an automated fashion (**b**, **c**, **d**, **h**). Rab5 immunostainings are shown in Supplementary Fig. 3h. The percentage change in lysosomal abundance was quantified using 3D-reconstructions analysis of lysosomes (**e**, **f**, **i**, **j**). **k**, **l** Representative Z-stack projections showing increased lysosome staining in nutrient fed Rap1A+B-depleted HEK293A cells (**k**). Bar graph shows relative change in average LAMP2 intensity across three individual experiments for 57–72 cells. The ratio between perinuclear and peripheral lysosomes in nutrient fed Rap1A+B-depleted cells is quantified in **l**. The change in lysosome abundance is shown in Supplementary Fig. 4a, b. Cell boundaries are depicted in yellow and cell nuclei (DAPI) is shown in blue. Scalebars: **a**, **g**, **k** = 10 μm, **e**, **i** = 20 μm. The microscopic fields imaged were randomly selected. Experiments were repeated at least three times. In **b**, **d**, **h**, **l** *n* denotes the number of individual cells analyzed across three independent experiments and data are presented as mean values ± s.d. In **f**, **j**, **k** *n* denotes the number of individual experiments and data are presented as mean values ± s.e.m. The number of cells analyzed to quantify lysosome abundance is shown in Supplementary Fig. 14. n.s. = not significant (*P* > 0.05); Student’s *t*-test; two-sided, unpaired (**d**, **f**, **j**, **k**), one-way ANOVA with Tukey’s post hoc test (**b**, **h**, **l**). In **h** *p* = 0.8148 and 0.8994 for siRNA Ctrl vs Rap1A+B #1 and #2 peripheral lysosomes, and 0.5731 and 0.585 for siRNA Ctrl vs Rap1A+B #1 and #2 for perinuclear lysosomes, respectively. In **b** *p* = 0.9927 and 0.9819 for Ctrl vs Rap1 WT, peripheral and perinuclear fractions, respectively. ***P < 0.001, **P < 0.01 one-way ANOVA with Tukey’s post hoc test (**h**). See Source Data File for statistics source data.

The spatiotemporal organization and abundance of lysosomes is dictated by nutrient availability^16–22^. Upon acute amino acid starvation, peripheral lysosomes positioned distal to the nucleus are lost and overall lysosomal abundance decreases (Supplementary Fig. 2a, b)^18,23^. We considered whether Rap1 might limit basal mTORC1 activity through one of two mechanisms: the release of mTORC1 from lysosomes^3,4^ or the reorganization of lysosomal architecture^18^. mTOR dissociation from lysosomes was unaffected by expression of constitutive active Rap1-G12V in full growth conditions (Supplementary Fig. 2c, d) and Rap1-G12V isoforms did not interact with mTOR (Supplementary Fig. 2e), or the mTORC1 and mTORC2 scaffolds Raptor and Rictor (Supplementary Fig. 2f, g), in agreement with previous mTOR interaction studies^24^. However, in the same Rap1-G12V expressing cells, lysosomal organization was strikingly similar to amino acid starved cells, despite being grown in amino acid-rich media. These cells had markedly decreased peripheral LAMP1/LAMP2/CD63/Rab7+ lysosomes and a subsequent increase in perinuclear lysosomes (Fig. 3a–c, Supplementary Fig. 3a–g), with no changes in the localization of Rab5 positive early endosomes (Fig. 3d, Supplementary Fig. 3h). 3D-reconstruction of lysosomes from confocal images also demonstrated a significant reduction in overall lysosome abundance in Rap1-G12V expressing cells when compared to wild-type Rap1-expressing cells or control cells (Fig. 3e, f). Further supporting a loss of lysosomes in Rap1-G12V-expressing cells, a decrease in LAMP2 levels was also observed by immunoblotting, and this decrease was more pronounced when both Rap1A and Rap1B G12V-mutants were co-expressed, compared to Rap1A-G12V alone (Supplementary Fig. 3i). Together, these data suggest that increased Rap1 activity, either through amino acid starvation or G12V mutation, suppresses the peripheral localization and abundance of lysosomes. We next determined if Rap1 limits lysosomes in response to amino acid starvation. After 4 h of amino acid starvation, lysosomes were mostly absent at the cell periphery in control cells, while Rap1-depleted cells maintained the lysosomal distribution observed in full growth conditions (Fig. 3g, h). Furthermore, amino acid starvation did not reduce lysosome abundance in Rap1-depleted cells, which instead had increased abundance when compared to fed control cells (Fig. 3i, j). We therefore also asked if Rap1 limits lysosomal abundance in amino acid-fed cells and found that Rap1 depletion robustly increased overall lysosome abundance (Fig. 3i–k, Supplementary Fig. 4a–e), which was phenotypically reversed by simultaneous expression of wild-type Rap1 (Supplementary Fig. 4f, g). Importantly, the increase in lysosome abundance is not caused simply by hyperactivation of mTORC1 signaling that occurs in Rap1-depleted cells, as hyperactivation of mTORC1 activity by ectopic Rheb expression did not alter lysosome abundance (Supplementary Fig. 4h). Notably, the ratio between perinuclear and peripheral lysosomes remained unaffected by Rap1 depletion in growing cells, suggesting that Rap1 depletion does not coincide with an increase of a specific pool of lysosomes (Fig. 3l). In conclusion, these data indicate that in addition to the amino acid starvation response, Rap1 also restricts lysosomal abundance in growing cells, further arguing that Rap1 is a pivotal regulator of the lysosomal network.

To examine how Rap1 regulates lysosome abundance, we considered three mechanisms: (i) lysosome exocytosis^25^, (ii) TFEB-driven transcription of lysosome genes^26^ or (iii) lysophagy, where lysosomes are eliminated by macro-autophagy^27,28^. We reasoned lysosomal exocytosis is an unlikely mechanism because starvation and Rap1-G12V expression concentrate peripheral lysosomes to the perinuclear region, with considerably fewer lysosomes moving toward the plasma membrane (Supplementary Movies 1, 2, Fig. 3a, b). Rap1 also did not regulate lysosome abundance via TFEB, as TFEB nuclear localization (Supplementary Fig. 5a–c), or the expression of TFEB-stimulated genes *LAMP1*, *CTSA, GALNS, HEXA1* and *RRAGD/*RagD (Supplementary Fig. 5d, e), were not induced by Rap1-depletion. Furthermore, neither increased or decreased Rap1 activity induced macro-autophagy as observed during TFEB activation (Supplementary Fig. 5f–h)^29^. To test whether lysosomes were being eliminated through lysophagy, we blocked autophagosome biogenesis by expression of dominant negative ATG4B-C74A (Supplementary Fig. 6a)^27,30^ or the Vps34 inhibitor PIKIII (Supplementary Fig. 6d)^31^ during the course of starvation. Lysosome abundance was still decreased in ATG4B-C74A-expressing cells or PIKIII-treated cells upon starvation as observed in control cells (Supplementary Fig. 6b, c), suggesting that lysosomes are suppressed independently of canonical lysophagy.

### Rap1-suppression of lysosome abundance is dependent on lysosome degradative function

**Fig. 4:**
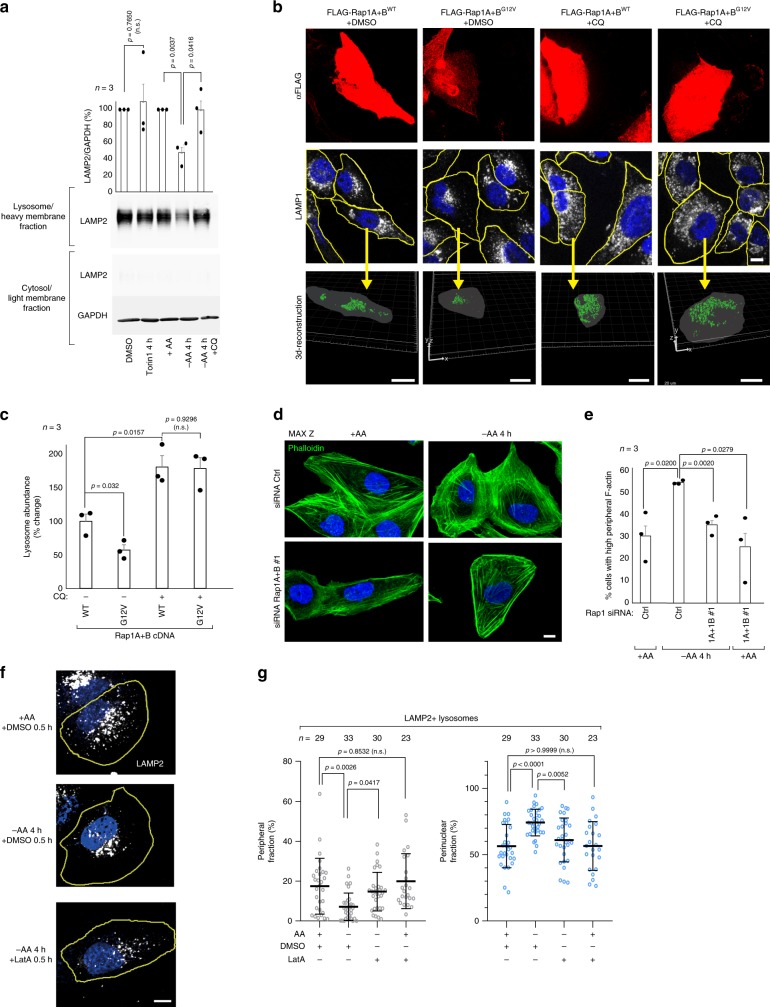
Lysosome degradative function is required for Rap1 suppression of lysosome abundance. **a** U2OS cells were subjected to 50 nM Torin1 or amino acid starvation in the presence or absence of 100 μM CQ for 4 h, and separated into lysosome-enriched heavy membranes and cytosolic fractions, and analyzed by immunoblotting for the indicated proteins. Cells were pretreated with CQ for 1 h before treatment was initiated. Graph shows the quantification of lysosome fraction from three individual experiments. **b**, **c** Representative Z-stack projections and 3d-reconstructions of the change in lysosomal abundance in U2OS cells transfected with the indicated FLAG-Rap1 cDNAs after treatment with 20 μM CQ for 24 h. 3d-reconstruction was performed on all FLAG-Rap1 cDNA expressing cells in the field of view. The yellow arrow indicates one representative cell for which the 3d-reconstruction is shown below. The percentage change in lysosomal abundance is quantified in **c**. The number of cells analyzed to quantify lysosome abundance is shown in Supplementary Fig. 14. Scale bars: middle panel 10 μm, lower panel 20 μm. **d**, **e** Amino acid starvation induces F-actin rearrangements in a Rap1-dependent manner, as shown in Rap1-depleted U2OS cells stained with Phalloidin (**d**), counting at least 300 cells per condition across three individual experiments and scoring for cells with increased peripheral F-actin (**e**). Scale bar: 10 μm. In **f**, **g** lysosomal distribution was assessed in U2OS cells that had been amino acid starved for 3.5 h and treated with either DMSO or 0.1 μM latrunculin A (Lat A) for an additional 30 min (total 4 h of starvation). Quantifications are shown in **g**. Also see Supplementary movie 16. Scale bar: 10 μm. The microscopic fields imaged were randomly selected. In **g** *n* denotes the number of individual cells analyzed across three independent experiments and data are presented as mean values ± s.d. In **a**, **c**, **e** *n* denotes the number of individual experiments and data are presented as mean values ± s.e.m. n.s. = not significant (*P* > 0.05); Student’s *t*-test; two-sided, unpaired (**a**, **c**, **e**), one-way ANOVA with Tukey’s post hoc test (**g**). See Source Data File for statistics source data. Uncropped images of blots are shown in Supplementary Fig. 17.

To better understand how Rap1 activation reorganizes lysosome architecture, we tracked mCherry-marked lysosomes over 4 h of amino acid starvation. In control cells, starvation induced a gradual re-localization of peripheral lysosomes to the perinuclear region and lysosomes appeared to reorganize into larger structures (Supplementary Fig. 7a, Supplementary Movies 1, 2). Occasionally, the lysosomal reorganization co-occurred with a change in cell shape, but the overall cell size did not change significantly in starved cells (Supplementary Fig. 7b). In contrast, Rap1-depleted cells seemed unable to reorganize their lysosome system when amino acids were limited (Supplementary Fig. 7a, Supplementary Movie 3). Even after 6.5 h of starvation, when starved control cells exhibited a striking decrease in the distance lysosomes tracked from the nucleus, Rap1-depleted cells appeared unaffected by amino acid withdrawal and did not appear to reorganize into larger structures (Supplementary Fig. 7a, c, d, Supplementary Movies 4–6). We considered whether the striking perinuclear accumulation of lysosomes plays a role in decreasing levels of lysosomes, and analyzed the ultrastructure of the perinuclear region of starved cells by transmission electron microscopy. Interestingly, perinuclear lysosomes/autolysosomes appeared larger and contained three-fold more intraluminal cargo than peripheral lysosomes/autolysosomes (Supplementary Fig. 8a–c). When lysosome acidification was blocked during the 4 h time course of starvation using the vacuolar H+-ATPase inhibitor concanamycin A (conA)^32^, we observed a marked increase in structures that resembled lysosomes in the lumen of perinuclear lysosomes/autolysosomes (Supplementary Fig. 9). Although the exact nature of these structures remains unclear, these results prompted us to investigate whether lysosomes are being eliminated in a lysosome-dependent manner when starved of amino acids. To this end, we assessed lysosomal abundance in starved cells where lysosome degradative function was blocked by conA or the lysomotrophic agent chloroquine (CQ)^32^. Strikingly, when lysosome function was inhibited, lysosome abundance was no longer suppressed by amino acid starvation and levels remained as observed in fed conditions (Fig. 4a, Supplementary Fig. 10a). We considered whether lysosome turnover was initiated by the loss of mTORC1 signaling upon starvation; however, no significant change in lysosome abundance (Fig. 4a) or organization (Supplementary Fig. 10b, c) was observed when mTORC1 was inhibited by Torin1 (Fig. 4a, Supplementary Fig. 10b, c) or rapamycin (Supplementary Fig. 10b, c) for 4 h. Since Rap1-G12V-expression decreases lysosome abundance, we next blocked lysosome degradation in these cells for 24 h with CQ or ConA. Similar to the results observed in amino acid-starved control cells, inhibition of lysosome function blocked the reduction in lysosome abundance by Rap1-G12V expression (Fig. 4b, c, Supplementary Fig. 10d, e), supporting a model that Rap1 promotes turnover of lysosomes in the perinuclear region in response to amino acid deprivation in a manner that requires continued lysosomal function. Notably, when lysosomal function was inhibited for 24 h, overall lysosome abundance increased in wild-type Rap1-expressing control cells (Fig. 4b, c, Supplementary Fig. 10d, e) or cells expressing mCherry-Lamp1 (Supplementary Fig. 10f, g), indicating that a basal rate of lysosomal turnover may exist in growing cells that can be modulated in a Rap1-dependent manner in addition to the TFEB-mediated induction in lysosome abundance observed under these conditions^33^.

### Rap1-perinuclear concentration of lysosomes is dependent on the actin cytoskeleton

To better understand how Rap1 concentrates lysosomes near the nucleus, we considered that Rap1 coordinates actin^10,34^, and that actin facilitates perinuclear localization of lysosomes^35^. When F-actin polymerization was inhibited with latrunculin A (latA), lysosome mobility was significantly increased (Supplementary Fig. 11a, b, Supplementary movies 7, 8), consistent with actin coordinating lysosome trafficking^36,37^. We analyzed F-actin dynamics in live cells marked by LifeAct^38^ and observed a pool of actin that dynamically associates with mCherry marked-lysosomes (Supplementary Fig. 11c, Supplementary movies 9, 10)^36,37^. To test whether Rap1 coordinates this pool of actin, we depleted cells of Rap1 and monitored the dynamics of lysosomes and F-actin in live cells. Interestingly, Rap1 depletion increased both actin mobility (Supplementary Fig. 11d, e, Supplementary movies 11, 12) and lysosomal mobility (Supplementary Fig. 11f, g, Supplementary movies 13, 14), suggesting that Rap1 is important for actin-mediated coordination of lysosomes. We tested whether the actin cytoskeleton is rearranged during amino acid starvation, when lysosomes move to the perinuclear region. Nearly twice the number of amino acid starved cells exhibited F-actin rearrangements, characterized by increased peripheral F-actin when compared to control cells (Fig. 4d, e, Supplementary movie 15), while no difference was observed in the organization of the microtubule cytoskeleton (Supplementary Fig. 12a). In contrast, F-actin was unaltered in Rap1-depleted cells (Fig. 4d, e), suggesting that these cells are unable to reorganize their actin cytoskeleton to drive lysosome perinuclear accumulation during amino acid starvation. To further test if actin mediates perinuclear accumulation of lysosomes upon starvation, we inhibited actin polymerization with latA in starved cells (Supplementary Fig. 12b, c) and observed lysosomes located in the perinuclear region migrating towards the cell periphery, adopting a lysosomal distribution resembling that of amino acid fed cells (Fig. 4f, g, Supplementary movie 16). This effect was also observed in Rap1-G12V-expressing cells upon actin inhibition (Supplementary Fig. 12d, e). Collectively, these data indicate that the Rap1-driven perinuclear organization of lysosomes is mediated through regulation of actin.

### Rap1 limits the lysosome surface available for mTORC1 activation

**Fig. 5:**
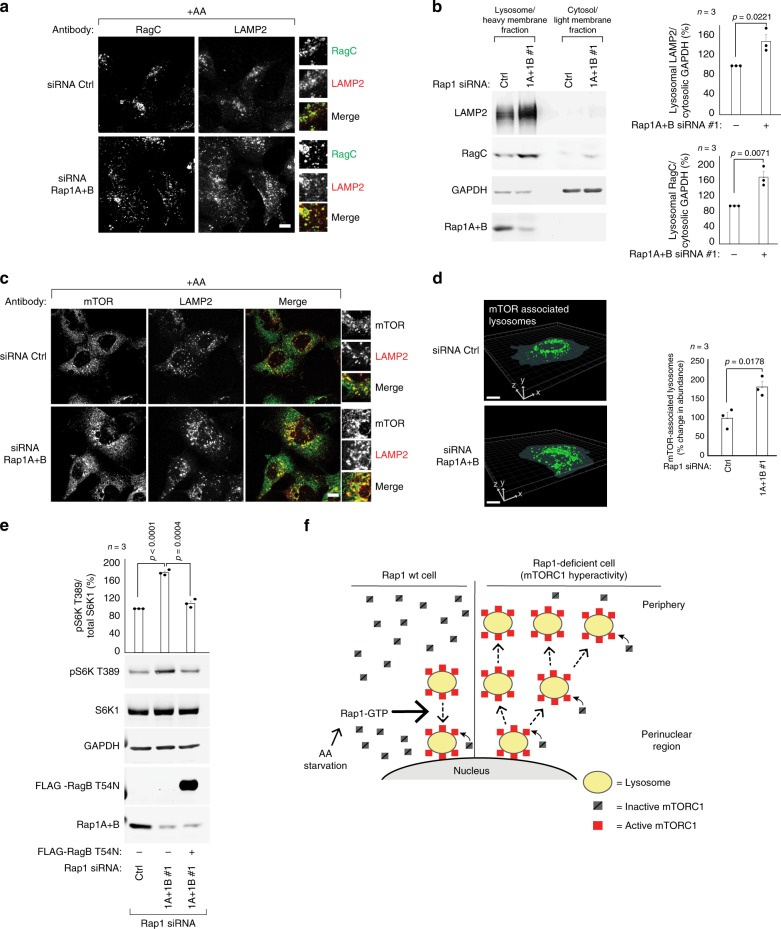
Rap1 depletion increases the interaction between mTORC1 and lysosomes. **a** Immunofluorescence images from a single confocal plane of Rap1A+B-depleted HEK293A cells. Samples were coimmunostained for RagC (green) and LAMP2 (red). **b** Lysates from control or Rap1A+B siRNA-depleted HEK293A cells were separated into lysosome-enriched heavy membranes and cytosolic/light membranes fractions, and analyzed by immunoblotting for the endogenous amounts of the indicated proteins. Graph shows quantification of lysosome fraction. **c**, **d** Samples were processed as in **a** and coimmunostained for mTOR (green) and LAMP2 (red). The relative change in mTOR-associated lysosomes was quantified using 3D-reconstruction analysis (**d**). The number of cells analyzed to quantify lysosome abundance is shown in Supplementary Fig. 14. **e** S6K1 phosphorylation in Rap1-depleted cells expressing dominant negative RagB-T54N cDNA to prevent mTOR localization to lysosomes. **f** Model: Rap1 suppresses lysosome abundance and peripheral expansion when amino acids are limited (left). When nutrient levels are sufficient, mTORC1 is recruited to and activated on lysosomes (left and right). Rap1 depletion leads to an aberrant increase in lysosomes and the surface available for mTORC1 activation. The enhanced association between mTORC1 and its lysosome-bound activators results in mTORC1 hyperactivity (right). Also, see Supplementary Fig. 13g. The microscopic fields imaged were randomly selected. All experiments were repeated at least three times and graphs represent relative immunoblot band intensity from *n* individual experiments. Statistical data are presented as mean values ± s.e.m; Student’s *t*-test; two-sided, unpaired. Scalebars:10 μm. Uncropped images of blots are shown in Supplementary Fig. 17. pS6K T389 and total S6K1 were processed on separate blots due to technical reasons. See Data Source File for statistics source data.

The lysosome surface functions as the platform for mTORC1 activation, where it is recruited by Rag-GTPases when nutrient levels are sufficient^3,4^. We reasoned that the Rap1-mediated changes in lysosome abundance alter the overall surface available for mTORC1 activation and asked if the increased population of lysosomes detected in Rap1-depleted cells are occupied by Rag-GTPases. In Rap1-depleted cells, RagC colocalized with LAMP2 to the same extent as control cells (Fig. 5a), arguing that these lysosomes carry RagC. Consistent with the increased abundance of lysosomes observed upon Rap1 depletion, we also isolated considerably more RagC associated with lysosome-enriched membranes from Rap1-depleted cells compared to control cells (Fig. 5b), further arguing that these cells contain more RagC-associated lysosomes. These results indicate that Rap1 controls the cellular capacity for mTORC1 activation: when Rap1 activity is increased during limitations in amino acids, the total lysosomal surface area decreases, and as a consequence so does mTORC1 activity. Conversely, when Rap1 is disrupted, the total Rag-populated lysosomal surface increases, resulting in elevated mTORC1 recruitment and hyperactivation (see model in Fig. 5f, Supplementary Fig. 13g). In support of this model, a marked increase in lysosomes containing mTOR was detected in Rap1-depleted cells compared to control cells by immunofluorescence imaging (Fig. 5c) and 3D-reconstruction analysis (Fig. 5d). Conversely, Rap1-G12V expression significantly reduced the pool of mTOR-bound lysosomes (Supplementary Fig. 13a, b). Notably, a surplus of mTOR is available in the cytoplasm for recruitment and activation at lysosomes when the abundance of these organelles increases (Supplementary Fig. 13c, Fig. 5c, Supplementary Fig. 2c)^39^. We, therefore, reasoned that disrupting recruitment of mTORC1 to the increased population of lysosomes in Rap1-depleted cells would reverse mTORC1 hyperactivity. In agreement with our model, mTORC1 hyperactivity in Rap1-depleted cells was reversed by dominant-negative RagB-T54N expression (Fig. 5e). Furthermore, mTORC1 signaling was still lost in Rap1-depleted cells subjected to amino acid starvation, despite having increased lysosome abundance, highlighting the requirement of the Rag-GTPase amino acid-sensing machinery in mTORC1 recruitment and activation (Supplementary Fig. 13d). To further test our model we increased lysosomal abundance by elevating TFEB expression^26^ (Supplementary Fig. 13e) which, in line with our reasoning, led to hyper-activated mTORC1 signaling equivalent to that observed in Rap1-depleted cells (Supplementary Fig. 13f, Fig. 1a). Collectively, these data argue that Rap1 suppresses mTORC1 activation during amino acid starvation by limiting the expansion of the lysosomal surface and consequently, loss of Rap1 leads to mTORC1 hyperactivity.

## Discussion

While it is established how lysosomes are replenished upon prolonged starvation^23,40^, the effects of acute amino acid starvation on lysosome homeostasis have remained less clear. Here, we report Rap1 as a critical regulator of the lysosome system in response to amino acid starvation. When amino acids are limited, Rap1 concentrates lysosomes in the perinuclear region, leading to a suppression in lysosome abundance. Although the mechanistic details of this starvation response require further investigation, including the identification of specific upstream and downstream Rap1 effectors, our data suggest that Rap1 promotes the turnover of lysosomes in a manner dependent on lysosome degradative function, but independent of canonical lysophagy. We speculate that such a form of lysosome turnover may involve engulfment of entire lysosomes, as observed previously^41^, or be restricted to the lysosome membrane^42^. Our data suggest that this Rap1-driven starvation response, which is independent of mTORC1 signaling, precedes processes that aim to replenish lysosomes, which are mediated by TFEB activation and lysosomal reformation in a mTORC1-regulated manner^23,33^. Rap1 appears to initiate this response by concentrating lysosomes in the perinuclear region through regulation of actin, although how individual Rap1 isoforms and effectors cooperate to achieve this regulation remains unclear; we speculate that the unique C-termini of the Rap1 isoforms generate differences in effector binding, localization or post-translational modifications, in line with the previous studies^43^. We find no evidence that Rap1 regulates the abundance of a distinct pool of lysosomes (Fig. 3l), yet it is possible that a particular lysosomal pool is responsible for mTORC1 activation, as suggested by others^18,44^. Importantly, we propose that lysosome abundance must be correctly coordinated with the nutrient status of cells, as an aberrant increase in the number of lysosomes leads to hyperactivated mTORC1. Thus, we put forward a model that the overall surface available for the mTORC1 activation machinery controls mTORC1 signaling output, in addition to the regulation of the activity, localization and protein levels of this machinery^3,45^. These data further highlight the importance of lysosomes in mTORC1 activation and point to a fundamental requirement of cells to maintain lysosome homeostasis.

## Methods

### Cell lines and tissue culture

HELA, HEK293T, U2OS, mouse embryonic fibroblasts (MEF) cells deficient in GCN2 and their littermate-derived wild-type control cells were obtained from ATCC. HEK293A cells were purchased from Thermo Fisher Scientific. RagA/B CRISPR/Cas9 knockout cells and control cells were kindly provided by Drs. Kun-Liang Guan and Jenna L. Jewell (University of California San Diego^46^). Mouse embryonic fibroblasts deficient in p14/LAMTOR2 and wild-type control cells were kindly provided by Drs. David Sabatini (Whitehead Institute) and Brendan Manning (Harvard School of Public Health). U2OS cells stably expressing mCherry-LAMP1 were generated through transfection of pLAMP1-mCherry and selection was carried out with 2 mg/mL G418 disulfate (Caisson Lab) whereafter a single colony of expressing cells was isolated.

All cell lines were cultured in DMEM with 4.5 g/L glucose (Life Technologies) with 10% FBS (Sigma-Aldrich) and 1% penicillin–streptomycin (Thermo Fisher Scientific). For amino acid starvation experiments, DMEM with 4.5 g/L glucose containing all amino acids (control) or no amino acids (-AA) were purchased from the Memorial Sloan Kettering Cancer Center Media Core Facility (New York, NY) and combined with dialyzed FBS (Sigma-Aldrich).

### Antibodies

Western blot: The following antibodies were purchased from Cell Signaling Technologies and used in a 1:1000 dilution: phospho p70 S6K1Thr389 (9234), S6K1 (2708), phospho 4E-BP1 Ser65 (9451), mTOR (2983), phospho Akt Ser473 (4060), Akt1 (2967), COX IV (4850), phospho PRAS40 Thr246 (2997), Rap1A+B (2399), Rap1B (2326), phospho AMPKalpha Thr172 (2535), LAMP1 (9091), AMPKalpha (2603), RagA (4357), RagC (3360), RagD (4470), Raptor (2280), p14/LAMTOR2/ROBLD3 (8145). Additional antibodies used in 1:1000 dilution: phospho GCN2 Thr899 (Abcam: ab75836), GCN2 (Novus Biologicals: MAB6878), LAMP2 (Santa Cruz Biotechnology: sc-18822), TFEB (Bethyl Laboratories, A303-673A), HA-probe (Santa Cruz Biotechnology: sc-805), LC3B (Novus Biologicals: NB100-2220), Calnexin (Abcam: ab75801), B-act (Abcam: ab8226). Antibodies used in 1:5000 dilutions: Anti-FLAG M2 (Sigma-Aldrich: F3165), GAPDH (Sigma-Aldrich: G8795).

In Supplementary Fig. 1b, the following horseradish peroxidase linked secondary antibodies were purchased from GE Healthcare and used in 1:5000 dilutions: Anti-Mouse igG (NXA931V) and Anti-Rabbit IgG (NA934V). For all other western blots, secondary antibodies were purchased from LI-COR Biosciences and used in 1:10,000 dilutions: Donkey Anti-Mouse IgG IRDye 680LT (926-68022), Donkey Anti-Rabbit IgG Antibody IRDye 800CW (926-32213).

Immunofluorescence: The following antibodies were used in 1:100 dilutions (validation refers to demonstration that immunolabeling is lost with siRNA knockdown or knockout of the antigen of interest): LAMP1 (Cell Signaling Technologies, 9091, validated in this study), LAMP2 (Santa Cruz Biotechnology, H4B4 sc-18822, validated in this study), mTOR (Cell Signaling Technologies, 2983, validated in this study and in ref. ^47^), CD63 (H5C6, BD Bioscience, validated in ref. ^48^), RagC (Cell Signaling Technologies, 3360), DYKDDDDK Tag (Cell Signaling Technologies, 14793), Rab7 (Cell Signaling Technologies, 9367, validated in this study and in ref. ^49^), a-tubulin (Sigma-Aldrich: T6199), Rab5 (Cell Signaling Technologies, 3547). LC3B (Cell Signaling Technologies, 3868), Anti-FLAG M2 (Sigma-Aldrich: F3165) was used in a 1:800 dilution and DAPI (Biotium) at a final concentration of 1 μg/mL. A detailed immunofluorescence procedure can be found below.

### siRNA

siRNAs were purchased from Sigma-Aldrich: Rap1A #1 (Hs01_00040405), Rap1A #2 (SASI_Hs02_00305929, targets the 3′UTR), Rap1B #1 (SASI_Hs02_00305940), Rap1B #2 (SASI_Hs02_00305936), Rap1B # 3 (SASI_Hs02_00305941, targets the 3’UTR), Rab7 (SASI_Hs01_00104357), LAMP1 siRNA (SASI_Hs02_00339819), LAMP2 siRNA (SASI_Hs01_00113171), universal negative control siRNA (SIC001), mTOR #1 (SASI_Hs01_00203145), mTOR #2 (SASI_Hs01_00203144).

### Reagents

Torin1 (Tocris Bioscience, 4247) was used at a final concentration of 50 nM and Rapamycin (Calbiochem, 553210) at a final concentration of 20 nM. Phalloidin (Thermo Fisher Scientific, A12379) was used according to the manufacturers’ recommendations. Latrunculin A was purchased from Tocris and used at a final concentration of 0.1 μM. PIK-III (Selleck Chemicals)^31^ was used at a final concentration of 5 μM, Concanamycin A (Cayman Chemical Company) at 100 nM, and Chloroquine (Sigma-Aldrich) at 100 μM or 20 μM, as specified in the figure legends.

### Plasmids

Human *RAP1A* and *RAP1B* cDNA was cloned into the pK-FLAG plasmid or pEGFP-C3 plasmid (Clonetech), generating N-terminal tagged expression constructs. *RAP1* G12V point mutations were introduced by site-directed mutagenesis (Agilent Technologies, 200521), using the following mutagenesis primers: *RAP1A* G12V for: CTAGTGGTCCTTGGTTCAGTAGGCGTTGGGAAGTCTGC, *RAP1A* G12V rev: GCAGACTTCCCAACGCCTACTGAACCAAGGACCACTAG, *RAP1B* G12V for: CTAGTCGTTCTTGGCTCAGTAGGCGTTGGAAAGTCTGC, *RAP1B* G12V rev: GCAGACTTTCCAACGCCTACTGAGCCAAGAACGACTAG. CFP_Gem_pcDNA4_HisMaxC was a gift from Henry Colecraft (Addgene plasmid # 41653^50^) and was cloned into the pK-FLAG plasmid. Dominant-negative RagB T54N, RagA QL, Rag C SN, mTOR-au1, Rheb, Rictor and Raptor expression constructs have been described previously^47,51–53^. pLAMP1-mCherry was a gift from Amy Palmer (Addgene 45147^54^) and TFEB-EGFP was kindly provided by Drs. Lewis Cantley and Mark Lundquist (Weill Cornell Medicine). mEGFP-Lifeact-7 was kindly provided by Michael Davidson (Addgene plasmid 54610). The mStrawberry-ATG4B-C74A construct^30^ was a kind gift from Drs. Fahad Benthani and Yan Feng (MSKCC).

### Transfections

Transfection of DNA and siRNA was performed using Lipofectamine 2000 (Thermo Fisher Scientific) according to the manufacturer’s protocol. DNA transfections of U2OS cells, using the GenJet In Vitro DNA Transfection Reagent (Ver. II, SL100489, SignaGen Laboratories), were performed according to the manufacturer’s protocol. Typically, cells were seeded one day before transfection and lysates prepared 26–30 h post transfection. In Fig. 1c, d, cells were lysed 36 h post transfection. DNA transfections of HEK293A cells were performed with 5.25 μg DNA per 10 cm plate, 0.875 μg DNA per 6-well, 0.35 μg DNA per 12-well and 0.175 μg DNA per 24-well; HEK293T with 1.5 μg DNA per 6-well; U2OS cells with 1 μg DNA per 6-well and 0.375 μg DNA per 24-well. siRNA transfections were performed with 600 pmol siRNA per 10 cm plate, 100 pmol siRNA per 6-well, 40 pmol siRNA per 12-well and 20 pmol siRNA per 24-well. In rescue experiments shown in Supplementary Fig. 1h and Supplementary Fig. 4f, g cells were transfected with wild-type Rap1A+B DNA or empty vector on day 1, transfected with siRNA targeting the 3′ UTR of Rap1A+B or control siRNA on day 2, and assessed on day 3. In Supplementary Fig. 5a–c and Supplementary Fig. 11d, e, cells were transfected with Rap1A+B siRNA in the morning and TFEB-EGFP or LifeAct-EGFP cDNA in the evening and assessed 24–30 h later. To prevent de-attachment of HEK293A and HEK293T cells, plates were treated with 31 μg/mL fibronectin (Corning) in PBS 1 h in room temperature before seeding.

### Cell lysates and immunoprecipitation

For immunoblotting, cells were washed once with ice-cold PBS and lysed on ice with immunoblotting lysis buffer containing 10 mM KPO_4_, 1 mM EDTA, 5 mM EGTA, 10 mM MgCl_2_, 0.5% NP-40, 0.1% Brij-35, 0.1% deoxycholate, 1 mM sodium vanadate, 50 mM beta-glycerophosphate, 400 μM PMSF, 0.02 μg/μL Leupeptin, 0.1 μg/μL pepstatin A, and 0.02 μg/μL aprotinin. Lysates were collected by centrifugation in a table-top centrifuge at 18,000 × *g* at 4 °C for 20 min whereafter protein concentrations were determined using the DC Protein Assay Kit II (Bio-Rad). Samples were boiled for 5 min in sample buffer with 5% 2-mercaptoethanol. 25–50 μg of lysate was resolved by SDS-PAGE and transferred to 0.2 μm nitrocellulose membranes (PerkinElmer), after which membranes were blocked with Odyssey buffer (LI-COR Biosciences) for 1 h. Membranes were incubated with primary antibodies over night at 4 °C, washed 3 times for 5 min with Tris-buffered saline containing 0.1% Tween 20 (TBST), incubated with secondary antibodies for 1 h in room temperature and washed 3 times for 15 min. Imaging was carried out using an Odyssey CLx Infrared Imaging System (LI-COR Biosciences), except for in Supplementary Fig. 1b, where images were captured with an Amersham Imager 680 (GE Healthcare), using SuperSignal West Pico PLUS Chemiluminescent Substrate from Thermo Scientific.

Quantifications of images were carried out in Image Studio (V. 5.2) (LI-COR Biosciences) according to the manufactures’ recommendations. At least three individual experiments were quantified.

For immunoprecipitation, cells were washed once with ice-cold PBS and lysed in buffer containing 40 mM HEPES pH 7.5, 120 mM NaCl, 1 mM EDTA, 5 mM MgCl_2_, 0.2% CHAPS, 10 mM pyrophosphate with protease and phosphatase inhibitors, after which lysates were centrifuged at 18,000 × *g* for 20 min at 4 °C in a table-top centrifuge. For each reaction, 40 μl of gel suspension of ANTI-FLAG M2 Affinity gel (Sigma-Aldrich) was used. Resins were washed three times before lysates were added. Samples were incubated for 2 h at 4 °C with rotation, whereafter resins were washed three times with lysis buffer and protein recovered by boiling samples for 5–10 min in sample buffer with 5% 2- mercaptoethanol.

To measure Rap1 activity, near confluent cells grown in 10 cm plates were washed once in ice-cold PBS and lysed, whereafter GTP-loaded Rap1 was isolated by immunoprecipitation, using the Rap1 binding domain (RBD) of Ral GDS from EMD-Millipore (14–455) or Cell Signaling Technologies (8818), according to the manufacturers protocol. The assay was validated per the manufacturer’s instructions.

Lysosome enriched heavy membranes were isolated from one-to-three 10 cm plates of nearly confluent cells and subjected to cell fractionation analysis, as previously described^55^. Briefly, cells were washed in ice-cold PBS and pelleted at 800 × *g* for 2 min at 4 °C, whereafter cells were resuspended in 10 mM HEPES pH 7.2, 10 mM KCL, 1.5 mM MgCl2, 250 mM sucrose, 1 mM sodium vanadate, 50 mM beta-glycerophosphate, 400 μM PMSF, 0.02 μg/μL Leupeptin, 0.1 μg/μL pepstatin A, and 0.02 μg/μμL aprotinin, and lysed by drawing lysates 4 times through a 23G needle. Next, lysates were centrifuged at 500 × *g* for 10 min, yielding a cytosolic supernatant lacking nuclei, which was further centrifuged at 20,000 × *g* for 2 h, thereby enriching the lysosome heavy-membranes in the pellet and the cytosolic/light membranes in the supernatant. Pellets were resuspended in the immunoblotting lysis-buffer. Protein concentrations were determined for the cytosolic fractions which both the cytosolic and the heavy membrane fractions were normalized to. Samples were analyzed by SDS-PAGE and immunoblotting. In Supplementary Fig. 1b, lysosomes were enriched using the Thermo Scientific Lysosome Enrichment Kit according to the manufacturers' protocol and a Beckman Optima LE-80K Ultracentrifuge.

All experiments were repeated at a minimum of three times unless otherwise specified in the figure legends.

### Amino acid starvation

Nearly confluent cultures were rinsed three times with amino acid free media containing dialyzed FBS. Cells were starved between 1 and 7.5 h in amino acid free media with 10% dialyzed FBS. HEK293T cells were serum deprived overnight before subjected to amino acid starvation and, where indicated, stimulated with amino acids for 30 min before cell lysis.

### Immunofluorescence

For fixed samples, 25000–30000 cells were sparsely seeded on cover slips in 24 wells. For HEK293A cells, cover slips were fibronectin coated prior to plating cells. Cells were fixed with 4% paraformaldehyde in PBS for 15 min at room temperature, whereafter cover slips were rinsed three times with PBS and permeabilized for 10 min in PBS with 0.2% Triton X-100. Samples were blocked for 1 h using a mixture of 50% Odyssey Blocking Buffer (LI-COR Biosciences) and 50% PBS with 0.2% Triton X-100. Samples were incubated with primary antibodies overnight at 4 °C. The following day, the cover slips were washed three times for 5 min with PBS and incubated for 1 h with Alexa488 or Alexa555 conjugated antibodies (Thermo Scientific) in 1:1000 dilutions. After washing three times for 10 min with PBS, samples were mounted using Dako Fluorescence Mounting Medium (Agilent Technologies).

Confocal images were captured with a Zeiss LSM 880 Laser Scanning Confocal Microscope, using appropriate bandpasses for fluorescence detection and 40X or 63X objectives with 1×–6x scanner zoom. The microscopic fields imaged were randomly selected. DAPI was excited at λ = 405 nm and detected at λ = 415–474 nm. Alexa-488 was excited at λ = 488 nm and detected at λ = 493–553 nm. Alexa-555 was excited at λ = 561 nm and detected at λ = 568–758 nm. Z-stacks were imaged with a z-step size of 0.45 μm. Live confocal cell images shown in Supplementary Fig. 7c and Supplementary Fig. 11 and Supplementary Movies 4–14 were captured at a single plane, using the 63X objective with 1.3–6x scanner zoom. Live images shown in Supplementary Fig. 7a and Supplementary Movies 1, 2, 3, 15, 16 were captured on a Nikon Eclipse Ti microscope equipped with an Andor Zyla sCMOS camera, using the 60x oil immersion objective. Live imaging experiments were carried out on a heated stage at 37 °C with 5% CO_2_, using 35 mm glass bottom dishes (#1.5 cover slips) with 20 mm micro-wells (CellVis).

In live cell imaging experiments with cells expressing mCherry-Lamp1 labeled lysosomes and LifeAct-labelled F-actin, acquisition settings were chosen that optimized tracking of lysosomes and actin for individual cells. In all other imaging-based experiments, images were always captured using identical settings across the experiment. Representative images are shown at the same magnification and using identical acquisition and display settings.

### Quantification of confocal images

For endosomal distribution analysis, confocal images at 40x magnification of sparsely seeded cells were captured as described above. Cells expressing FLAG-tagged Rap1 cDNA were stained with anti-FLAG (to identify cDNA expressing cells) and LAMP1. siRNA transfected cells were stained with LAMP2 and Alexa Fluor 488 Phalloidin (Thermo Fisher Scientific) to visualize cell boundaries. The endosomal distribution was quantified in an automated way using ImageJ/Fiji. Briefly, max z-projections and binary images of the nucleus, lysosomes and cell area were generated for individual cells using thresholding and settings that was appropriate for control samples, and these settings were used throughout the analysis of each experiment. The cell body was segmented into two areas: one containing the perinuclear area and one containing the peripheral area. The perinuclear area was defined to be within 5 μm of the nucleus and the peripheral area as more than 10 μm from the nucleus. The fraction of endosomes within the peripheral and perinuclear area was quantified for individual cells across at least three individual experiments. Examples of binary images and a cell where the regions for the perinuclear and peripheral have been generated are shown in Supplementary Fig. 3b, c. Average pixel intensity and cell size was determined in Fiji/ImageJ. In Fig. 3k, the average LAMP2 intensity was determined for a total of 57–72 cells across three individual experiments. The intensity was normalized to control conditions within individual experiments and are presented as percentage change in LAMP2 intensity. In Supplementary Fig. 10f, g, the number of lysosomes was quantified for individual cells using images from live confocal experiments of U2OS-mCherry-Lamp1 cells captured on a single z-plane for 30–60 s. Lysosome number was normalized to the total cell area and this was repeated for multiple cells across three individual experiments.

For 3D-reconstruction analysis of lysosome abundance, confocal images of LAMP1 or LAMP2 and mTOR coimmunostained cells were captured at 63X with 1.6x scanner zoom, using a z-step size of 0.45 μm. 3D-reconstructions of lysosomes and mTOR-associated lysosomes were created in Imaris (Bitplane, v. x64 8.3.1) using appropriate thresholds for controls. For individual or multiple cells, the combined lysosomal volume was normalized to the total volume of the cell(s), and this was repeated for multiple cells across at least three individual experiments. As immunoreactivity differs slightly between individual experiments, data was normalized to control conditions within individual experiments. Supplementary Fig. 14d specifies the number of cells analyzed across at least three individual experiments.

To quantify F-actin distribution, images of sparsely seeded Rap1A+B siRNA depleted cells or control cells that had been subjected to amino acid deprivation and stained with Phallodin were scored into three categories: cells containing high/dense peripheral F-actin, cells that did not contain high/dense peripheral F-actin or cells that could not be scored, for example, because cells were too densely populated in an area of an image. The scorer was blinded to treatment conditions represented in the images and over 380 cells were counted across three individual experiments.

Vesicle trafficking was analyzed using TrackMate (v3.4.0) for Fiji/ImageJ, using the DoG detector, vesicle diameter = 1 μm with the appropriate settings and thresholds for control samples. The average vesicle displacement was calculated for multiple cells across three individual experiments.

Displacement of actin in Supplementary Fig. 11d, e was quantified manually using Fiji/ImageJ. Briefly, the change in displacement was measured for a minimum of 122 actin patches per condition over 60 s by a scorer that was blinded to treatment, analyzing at least 10 cells across three individual experiments.

Co-localization percentages were determined in Imaris (Bitplane, v. x64 8.3.1) for confocal images captured at 63X with 1.6x scanner zoom. Manders’ co-localization coefficient of the mTOR signal that co-localized with the lysosome signal were determined for individual cells across three independent experiments, using automatic Costes thresholding.

### Statistical analysis

All experiments were repeated at least three times except for electron microscopy analyses, which were repeated a total of two times. For statistical analysis, *p*-values were determined using two-sided Student’s *t* test or one-way ANOVA with Tukey’s post hoc test, as indicated in the figure legends. Also, see Source Data File for statistical source data.

### Electron microscopy analysis

Cells were washed with serum-free media or appropriate buffer then fixed with a modified Karmovsky’s fix of 2.5% glutaraldehyde, 4% paraformaldehyde and 0.02% picric acid in 0.1 M sodium cacodylate buffer at pH 7.2^56^. Following a secondary fixation in 1% osmium tetroxide, 1.5% potassium ferricyanide^57^, samples were dehydrated through a graded ethanol series, and embedded in situ in LX-112 resin (Ladd Research Industries). En face ultrathin sections were cut using a Diatome diamond knife (Diatome, USA, Hatfield, PA) on a Leica Ultracut S ultramicrotome (Leica, Vienna, Austria). Sections were collected on copper grids and further contrasted with lead citrate^58^ and viewed on a JEM 1400 electron microscope (JEOL, USA, Inc., Peabody, MA) operated at 120 kV. Digital images were captured on a Veleta 2 K × 2 K CCD camera (Olympus-SIS, Germany).

### qPCR

RNA extraction was accomplished using PureLink RNA Mini Kit (Life Technologies) and was subjected to DNase I treatment (Sigma-Aldrich), whereafter complementary DNA was synthesized from 1 mg of total RNA using iScript™ cDNA Synthesis Kit (Bio-Rad Laboratories) according to the protocol. Analysis was carried out on a QuantStudio 6 Flex (Applied Biosystems). Expression was normalized to *GAPDH* and the following primers were used, shown in 5’ to 3’: *RAP1A* for: CGTGAGTACAAGCTAGTGGTCC, *RAP1A* rev: CCAGGATTTCGAGCATACACTG, *RAP1B* for: AACAGATTCTTCGAGTTAAAGACACTGA, *RAP1B* rev: TTTGACCTTGTTCCTTCCCTACA, *GAPDH* for: ATGGGGAAGGTGAAGGTCG, *GAPDH* rev: GGGGTCATTGATGGCAACAATA, *CTSA* for: TCCCAGCATGAACCTTCAGG, *CTSA* rev: AGTAGGCAAAGTAGACCAGGG, *LAMP1* for: CACGAGAAATGCAACACGTTAC, *LAMP1* rev: GGGTGCCACTAACACATCTGTAT, *GALNS* for: GTGACCTCGGGGTGTATGGA, *GALNS* rev: AAGCCATTGCGGATGGGTAG, *HEXA* for: CAGGGAAACGGCATACACTGG, *HEXA* rev: TGGTCAGGGTATAATTCTCCACT, *RRAGD* for: AGAAGCGGCAAGTCGTCTATT, *RRAGD* rev: TTCCCGGCATATCTTATTAGTGC. Experiments were repeated at least three times. For statistical analysis, p-values were determined using two-sided Student’s t test. Statistical data are presented as mean values ± standard error of the mean.

### Reporting summary

Further information on research design is available in the Nature Research Reporting Summary linked to this article.

## Supplementary information

Supplementary Information

Description of Additional Supplementary Files

Supplementary Movie 1

Supplementary Movie 2

Supplementary Movie 3

Supplementary Movie 4

Supplementary Movie 5

Supplementary Movie 6

Supplementary Movie 7

Supplementary Movie 8

Supplementary Movie 9

Supplementary Movie 10

Supplementary Movie 11

Supplementary Movie 12

Supplementary Movie 13

Supplementary Movie 14

Supplementary Movie 15

Supplementary Movie 16

Reporting Summary

## Data Availability

All data supporting the findings of this study are available from the corresponding author upon reasonable request. Statistical source data for Figs. 1–5 and Supplementary Figs. 1–14 are provided in Source Data File.

## Source Data

Source Data

## Code availability

Custom Fiji/ImageJ Macro scripts that were generated to automate some of the image quantifications are available upon reasonable request.
