# Supplementary material for: Rap1-GTPases control mTORC1 activity by coordinating lysosome organization with amino acid availability
Source: Nat Commun. 2020 Mar 17;11:1416. doi: 10.1038/s41467-020-15156-5 (PMC7078236; doi:10.1038/s41467-020-15156-5)
Supplement: Supplementary file 1 — Supplementary Information [file 41467_2020_15156_MOESM1_ESM.pdf]

Supplementary Information

Rap1-GTPases control mTORC1 activity by coordinating lysosome organization with amino acid availability

Anders P. Mutvei, Michal J. Nagiec, Jens C. Hamann, Sang Gyun Kim, C. Theresa Vincent and John Blenis

Correspondence to: jblenis@med.cornell.edu, anders.mutvei@igp.uu.se

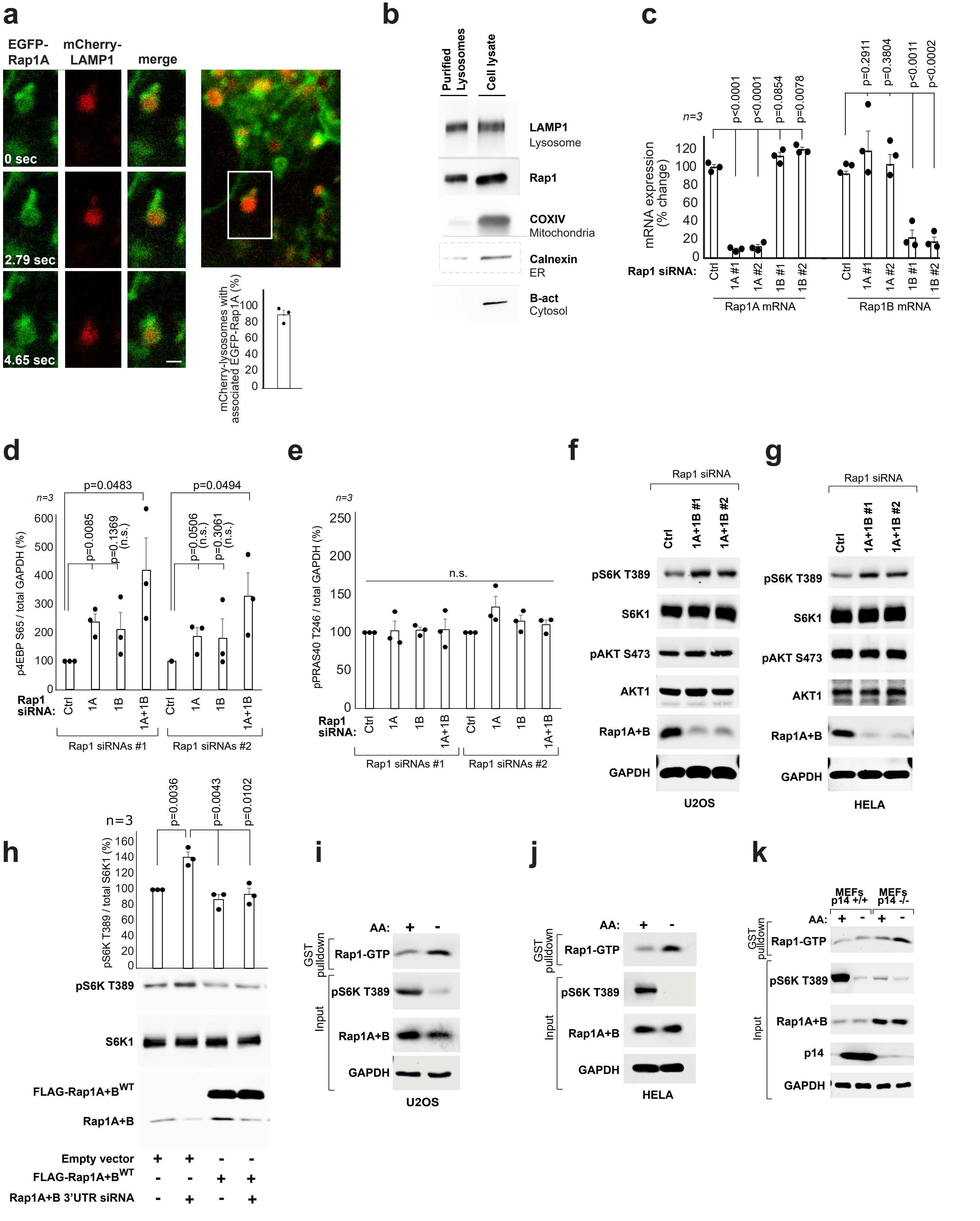

Supplementary Figure 1

Supplementary Fig 1. Rap1 localizes to lysosomes and suppresses mTORC1 signaling during limitations in amino acids, independently of p14-LAMTOR2.

(**a**) HEK293A cells were transfected with wild-type EGFP-Rap1A (green) or Lamp1-mCherry to mark lysosomes (red) and observed using live confocal microscopy. Time-lapse images (seconds) show Rap1A (green) associating with lysosomes (red). The graph displays the percentage of LAMP1-mCherry lysosomes found to be associated with wild-type EGFP-Rap1A; 202 lysosomes were quantified across three individual experiments (n=3). (**b**) Immunoblots showing that Rap1 is abundant in lysates of purified lysosomes. (**c**) Rap1A (1A) or Rap1B (1B) mRNA expression in HEK293A cells depleted of Rap1A or Rap1B, using two individual sets of siRNA for each isoform (siRNA #1 and #2) or control (Ctrl). (**d-e**) Quantifications of p4EBP S65 **(d)** and pPRAS40 T246 **(e).** Immunoblots are shown in Fig 1a. In (**f-g**), mTORC1 and AKT signaling levels in full growth conditions were determined for U2OS cells (**f**) or HELA cells (**g**)**,** by immunoblotting against the indicated endogenous proteins. **(h)** HEK293A cells were transfected with wild-type Rap1A+B on day 1 and siRNA targeting the 3'UTRs of Rap1A+B on day 2, whereafter S6K1 phosphorylation was assessed by immunoblotting on day 3. Rap1 was detected using a Rap1A+B antibody.
In (**i**) through (**k**), endogenous Rap1 activity (Rap1A+B-GTP) was assessed after 1 hour of amino acid starvation in U2OS cells (**i**), HELA cells (**j**) and mouse embryonic fibroblasts (MEFs) deficient for p14/Lamtor2 or corresponding isogeneic control cells (**k**). Scale bar: 1μm. All experiments were repeated at least three times except for in (**i**-**j**), where experiments were repeated two times. pS6K T389 and total S6K1, as well as p4EBP, pPRAS40 and GAPDH were processed on separate blots due to technical reasons. Graphs represent relative immunoblot band intensity from *n* individual experiments. n.s.= not significant ($P>0.05$); Student's *t*-test; two-sided, unpaired. Statistical data are presented as mean values, +/- s.e.m. See Source data file for statistics source data. Uncropped images of blots are shown in Supplementary Figures 17-18.

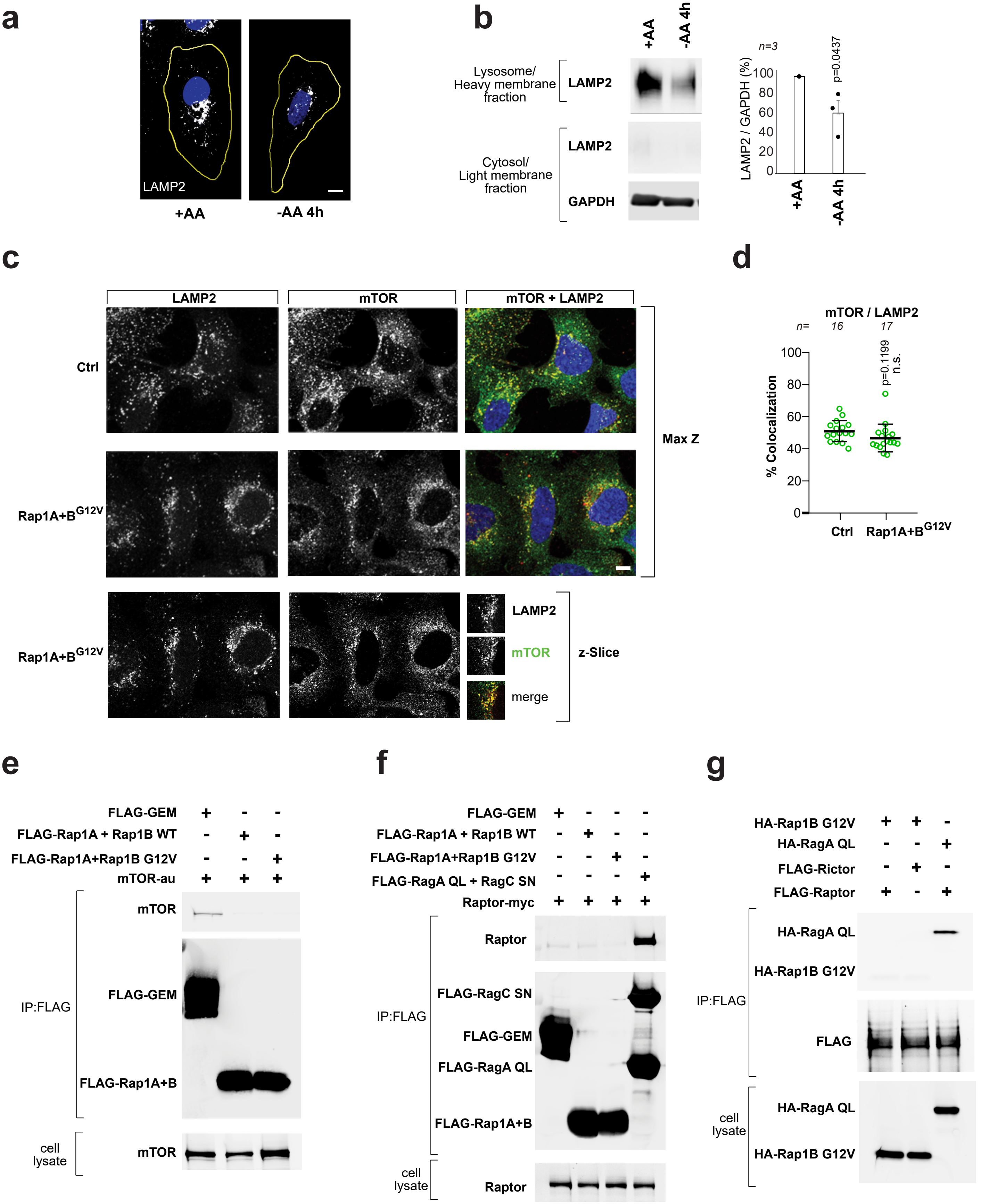

Supplementary Figure 2

Supplementary Figure 2. Rap1 activation does not promote the release of mTOR from lysosomes

(**a-b**) Representative z-stack projections **(a)** and immunoblots **(b)** of endogenous LAMP2 in U2OS cells starved of amino acids for 4 hours. **(c)** Representative confocal images of the endogenous distribution of LAMP2+ lysosomes (red) and mTOR (green) are shown after expressing cDNA for G12V Rap1A+B or empty vector control in HEK293A cells. Z-stack projections are shown in the upper panel (max z, upper panel) and single confocal planes in the lower panel (z-Slice, lower panel) with nuclear DAPI in blue. Percent mTOR colocalized with LAMP2 across three individual experiments is quantified in (**d**). In (**e**) to (**g**), HEK293A cells were transfected with the specified cDNA expression vectors, whereafter FLAG-immunoprecipitates were subjected to immunoblotting of the indicated proteins. All experiments were repeated at least three times except for in (**g**), where experiments were repeated two times. In **(d)**, *n* denotes the number of individual cells analyzed across 3 independent experiments and data are presented as mean values +/- s.d. In **(b)**, graph represent relative immunoblot band intensity from three individual experiments (n=3) and data are presented as mean values +/- s.e.m. n.s.= not significant; Student's *t*-test; two-sided, unpaired. Cell boundaries are depicted in yellow. The microscopic fields imaged were randomly selected. Scalebar: 10 μm. See Source Data file for statistics source data. Uncropped images of blots are shown in Supplementary Figures 18-19.

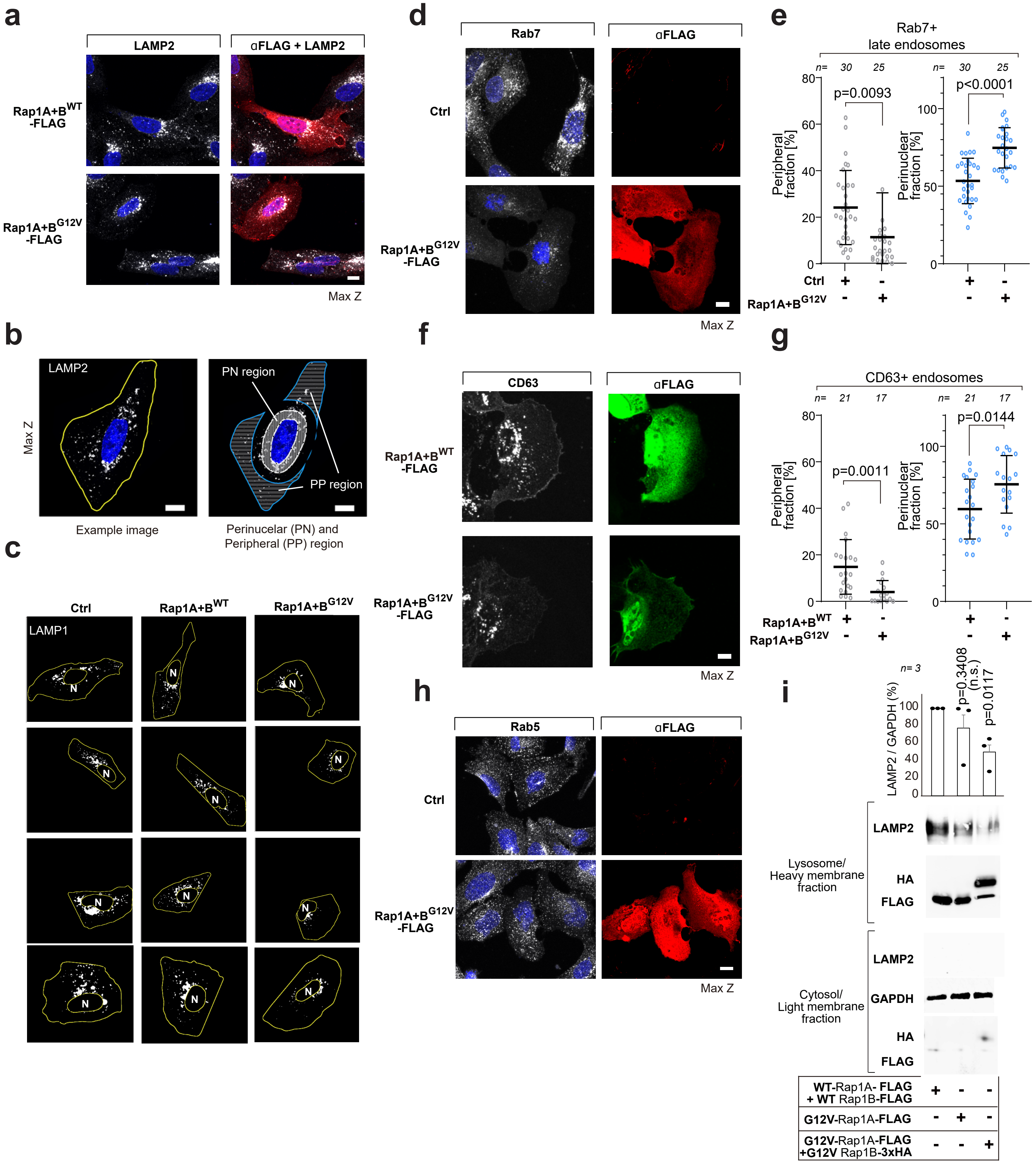

Supplementary Figure 3

Supplementary Figure 3. Endosomal distribution analysis of Rap1-expressing cells

(**a**) Representative z-stack projections of endogenous LAMP2 distribution in cells expressing FLAG-tagged wild-type Rap1A+B or G12V Rap1A+B. G12V-expressing cell is also shown in Fig 3a. (**b**) Example of a cell where the perinuclear and peripheral regions have been generated in an automated fashion in ImageJ/Fiji. **(c)** Binary images showing the LAMP1+ lysosome distribution in white for U2OS cells expressing FLAG-tagged cDNA for wild-type (wt) Rap1A+B, Rap1A+B G12V or control. Binary images were generated in an automated fashion in ImageJ/Fiji, from which the perinuclear and peripheral lysosome fractions were quantified. For details, refer to Materials and Methods.

(d-h) Representative z-stack projections of the endogenous endosomal distribution of Rab7 **(d)** CD63 **(f)** and Rab5 **(h)** are shown after expressing cDNA for wild-type Rap1A+B, G12V Rap1A+B or control (Ctrl) in U2OS cells, as indicated. Quantifications are shown in **(e)**, **(g)** and Fig 3d.

(i) HEK293A-cells were transfected with FLAG-tagged wild-type Rap1A+B, FLAG-Rap1A-G12V or FLAG-Rap1A-G12V + 3xHA-Rap1B-G12V, whereafter cell lysates were separated into lysosome-enriched heavy membranes and cytosolic/light membranes fractions, and analyzed by immunoblotting for changes in the amounts of indicated endogenous proteins.

Scalebars: 10 μm. Cell boundaries are depicted in yellow. Cell nuclei (DAPI) is displayed in blue. N=nuclei. The microscopic fields imaged were randomly selected. In **(e,g)**, *n* denotes the number of individual cells analyzed across 3 independent experiments and data are presented as mean values +/- s.d. In **(i)**, graph represent relative immunoblot band intensity from three individual experiments (n=3) and data are presented as mean values +/- s.e.m. n.s.= not significant; Student's *t*-test; two-sided, unpaired. Independent experiments were repeated at least three times. See Source Data File for statistics source data. Uncropped images of blots are shown in Supplementary Figure 19.

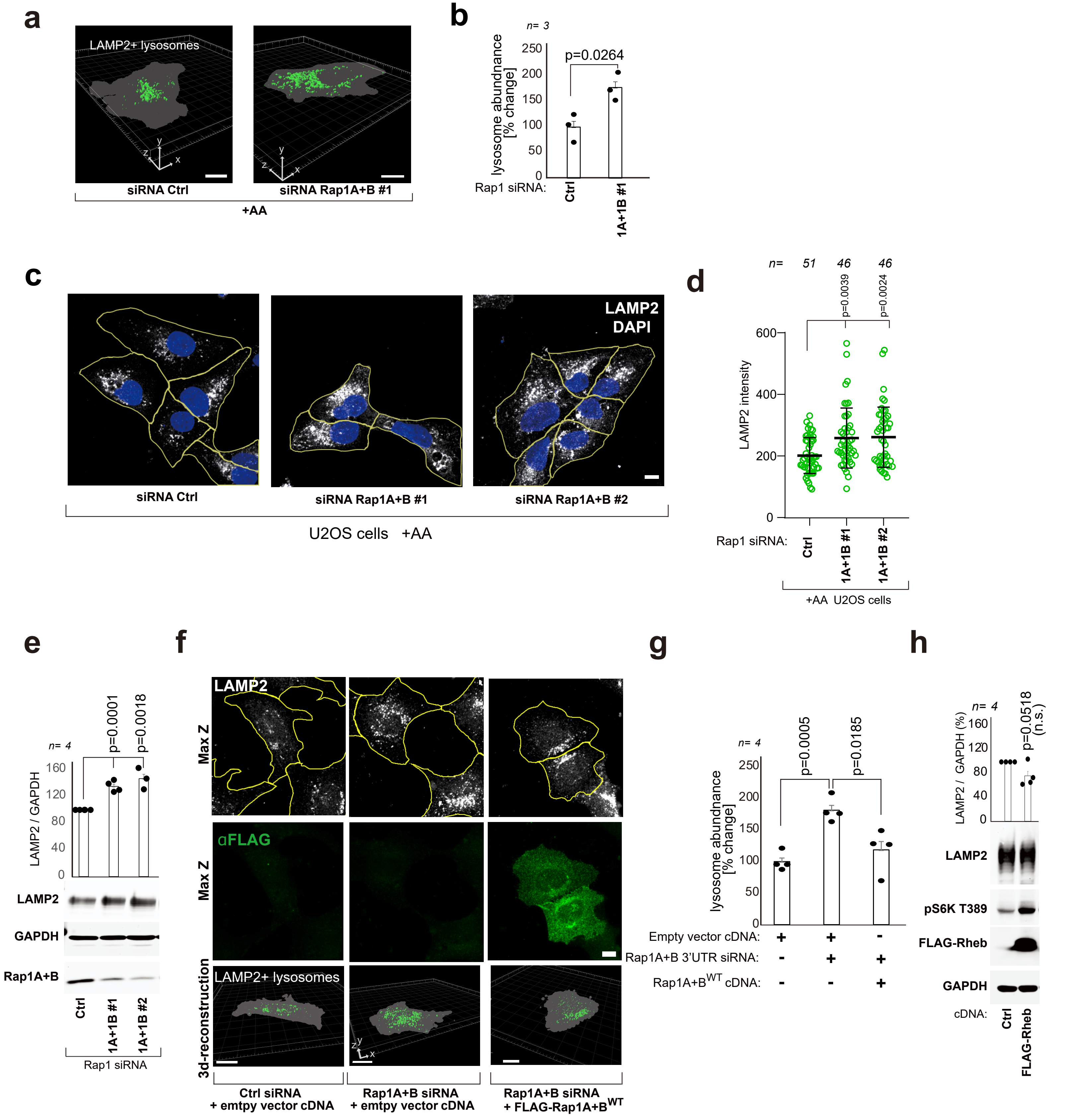

Supplementary Figure 4

Supplementary Figure 4. Rap1 depletion increases lysosome abundance in nutrient fed cells

(a-b) The change in in lysosome abundance in Rap1 depleted HEK293A cells or control cells is quantified. See Fig 3k for representative images. In (**c-d**), representative z-stack projections of the endogenous LAMP2+ lysosome staining in control cells or Rap1A+B siRNA depleted U2OS cells are shown. Cell nuclei (DAPI) is displayed in blue. Quantification of the average LAMP2 intensity from three independent experiments is shown in (**d**). (**e**) Immunoblot showing endogenous LAMP2 protein levels in HEK293A cells depleted of Rap1A+B using two sets of siRNA. **(f-g)** U2OS-cells were transfected with FLAG-tagged wild-type Rap1A+B on day 1 and siRNA targeting the 3'UTRs of Rap1A+B on day 2, whereafter the change in lysosome abundance was assessed on day 3. Representative z-stack projections of the endogenous LAMP2 distribution and representative 3D-reconstucions are shown in **(f),** with quantifications in **(g)**. (**h**) Immunoblot showing endogenous LAMP2 protein levels in HEK293A cells expressing FLAG-tagged wild-type Rheb. The number of cells analyzed in the quantification of lysosome abundance across three individual experiments is shown in Supplementary Figure 14. Scalebars: **(a,c)** 10 μm, **(f)** middle panel: 10 μm, lower panels: 20 μm. In **(d)**, *n* denotes the number of individual cells analyzed across 3 independent experiments and data are presented as mean values +/- s.d. In **(b,e,g,h)**, *n* denotes the number of individual experiments and data are presented as mean values +/- s.e.m. n.s.= not significant; Student's *t*-test; two-sided, unpaired (**b,e,g,h**) or one-way ANOVA with Tukey's post hoc test **(d).** The microscopic fields imaged were randomly selected. See Source Data File for statistics source data. Uncropped images of blots are shown in Supplementary Figure 19.

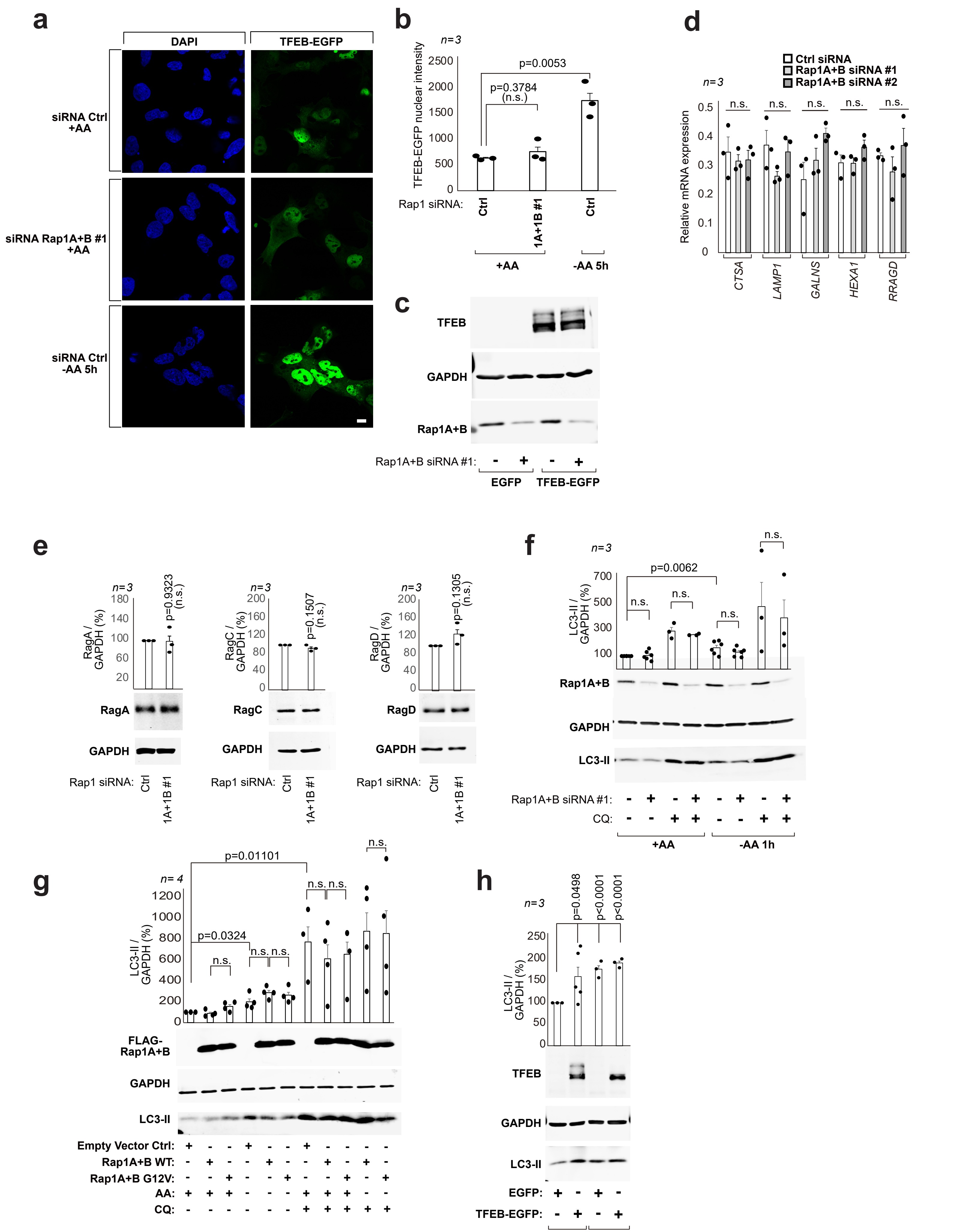

Supplementary Figure 5

Supplementary Figure 5. TFEB-signaling is not increased in Rap1-depleted cells.

(a) Images from a single confocal plane of Rap1A+B siRNA depleted HEK293A cells expressing EGFP-TFEB. **(b)** Average nuclear EGFP intensity quantified from three individual experiments with a total of 108-262 cells per condition. In **(c)**, lysates from **(a-b)** were immunoblotted for the indicated proteins.

(d) mRNA expression levels of the indicated TFEB-responsive genes were assessed in Rap1A+B siRNA depleted HEK293A cells by quantitative PCR. See Source Data file for exact p-values.

(e-h) Lysates from Rap1A+B siRNA depleted HEK293A cells **(e-f)** Rap1A+B WT or G12V expressing cells **(g)** or TFEB-EGFP expressing HEK293A cells **(h)** were subjected to immunoblotting against the indicated endogenous proteins. In **(f-h),** cells were starved for 1 hour of all amino acids and treated with 100 μM chloroquine (CQ), as indicated.

All experiments were repeated at least three times. LC3II and GAPDH were in some replicates processed on separate blots for technical reasons. The microscopic fields imaged were randomly selected. *n* denotes the number of individual experiments analyzed and data are presented as mean values, +/- s.e.m. n.s.= not significant ($P>0.05$); Student's *t*-test; two-sided, unpaired. In **(f)**, n.s. corresponds to the following p-values (from left to right): p=0.6981, 0.4611, 0.2081 and 0.7752. In **(g)**, n.s. corresponds to the following p-values (from left to right): p=0.0507, 0.1050, 0.6169, 0.5720, 0.8570 and 0.9544. See Source Data File for statistics source data. Uncropped images of blots are shown in Supplementary Figure 19-20.

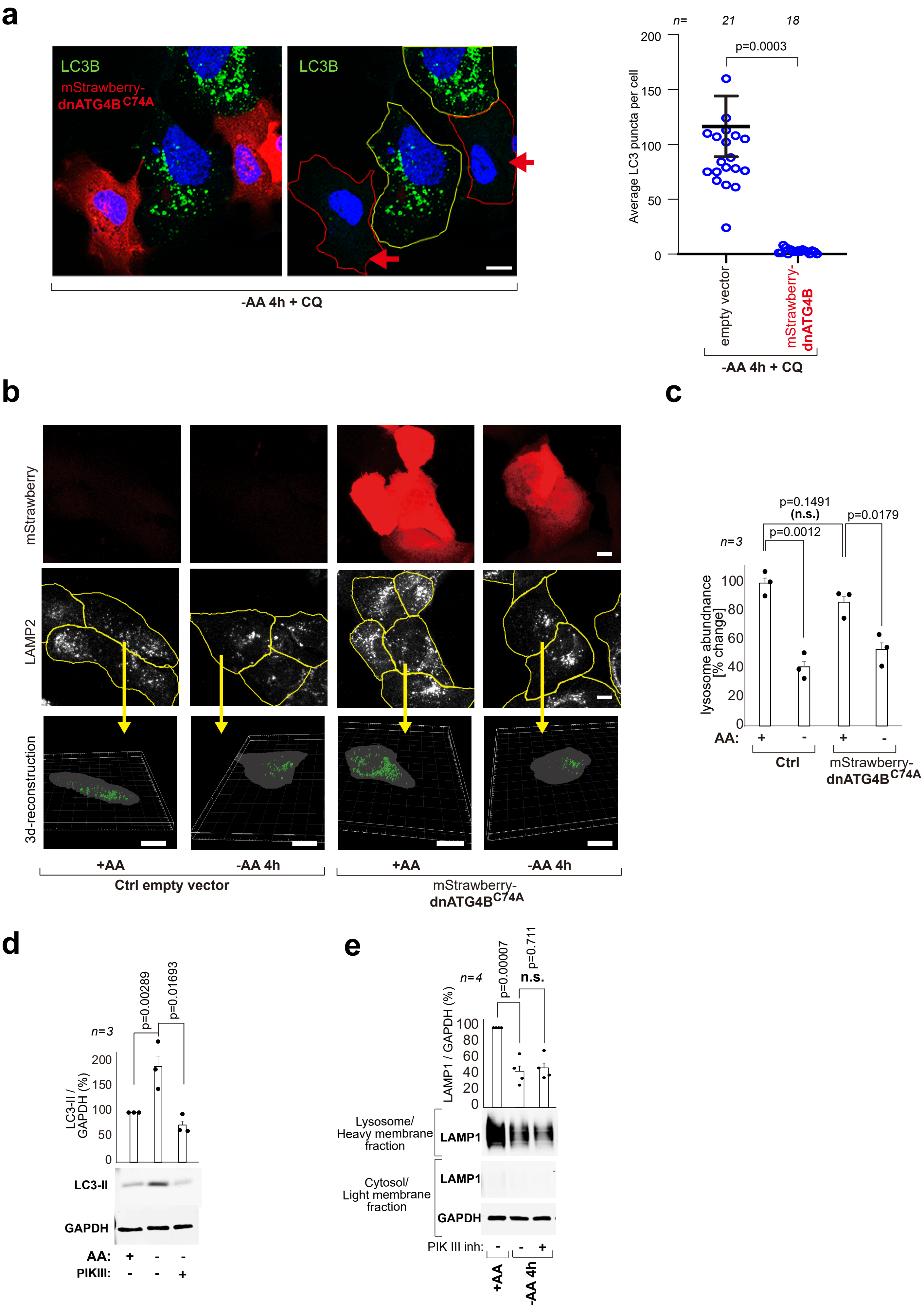

Supplementary Figure 6

Supplementary Figure 6. The suppression of lysosome abundance is independent of canonical lysophagy

(a) Representative images of the endogenous LC3B staining in U2OS cells where autophagosome biogenesis was inhibited by expression of dominant-negative (dn) mStrawberry-ATG4B C74A. To accumulate LC3B-positive autophagosomes, cells were starved of amino acids for four hours in the presence of 100 μM chloroquine (CQ). Average LC3B puncta per cell is quantified to the right. Red arrowheads indicate mStrawberry-dnATG4-C74A expressing cells. Cell boundaries are depicted in yellow for untransfected cells and red for dnATG4-C74A expressing cells. Scale bar: 10 μm.

(b-c) Lysosome abundance still decreases upon amino acid starvation in cells deficient in canonical autophagy. Representative Z-stack projections and 3d-reconstructions of the change in lysosomal abundance in U2OS cells expressing mStrawberry-dnATG4-C74A are shown in **(b)**, with quantifications in **(c)**. In control cells, 3d-reconstruction was performed on all cells within the field of view. In mStrawberry-dnATG4-C74A expressing cells, 3d-reconustunction was performed on all mStrawberry-positive cells in the field of view. The yellow arrow indicates one representative cell for which the 3d-reconstruction is shown below. The number of cells analyzed to quantify lysosome abundance is shown in Supplementary Figure 14. Scale bars: upper and middle panel: 10 μm, lower panel: 20 μm.

(d-e) Lysosome abundance still decreases upon amino acid starvation in cells where canonical autophagy is blocked by the VPS34 inhibitor PIK-III. Lysates were prepared from amino acid starved treated with DMSO control or 5 μM PIK-III for one hour (**d**) or four hours (**e**) and analyzed by immunoblotting for the endogenous amounts of the indicated proteins. In **(e)**, lysates were separated into lysosome-enriched heavy membranes and cytosolic/light membranes fractions. Cells were pretreated with PIKIII or DMSO for one hour before amino acid starvation was initiated. In **(a)**, *n* denotes the number of individual cells analyzed across three independent experiments and data are presented as mean values +/- s.d. In **(c,d,e)**, *n* denotes the number of individual experiments and data are presented as mean values +/- s.e.m. n.s.= not significant (P>0.05); Student's *t*-test; two-sided, unpaired**.** See Source Data File for statistics source data. Uncropped images of blots are shown in Supplementary Figure 21.

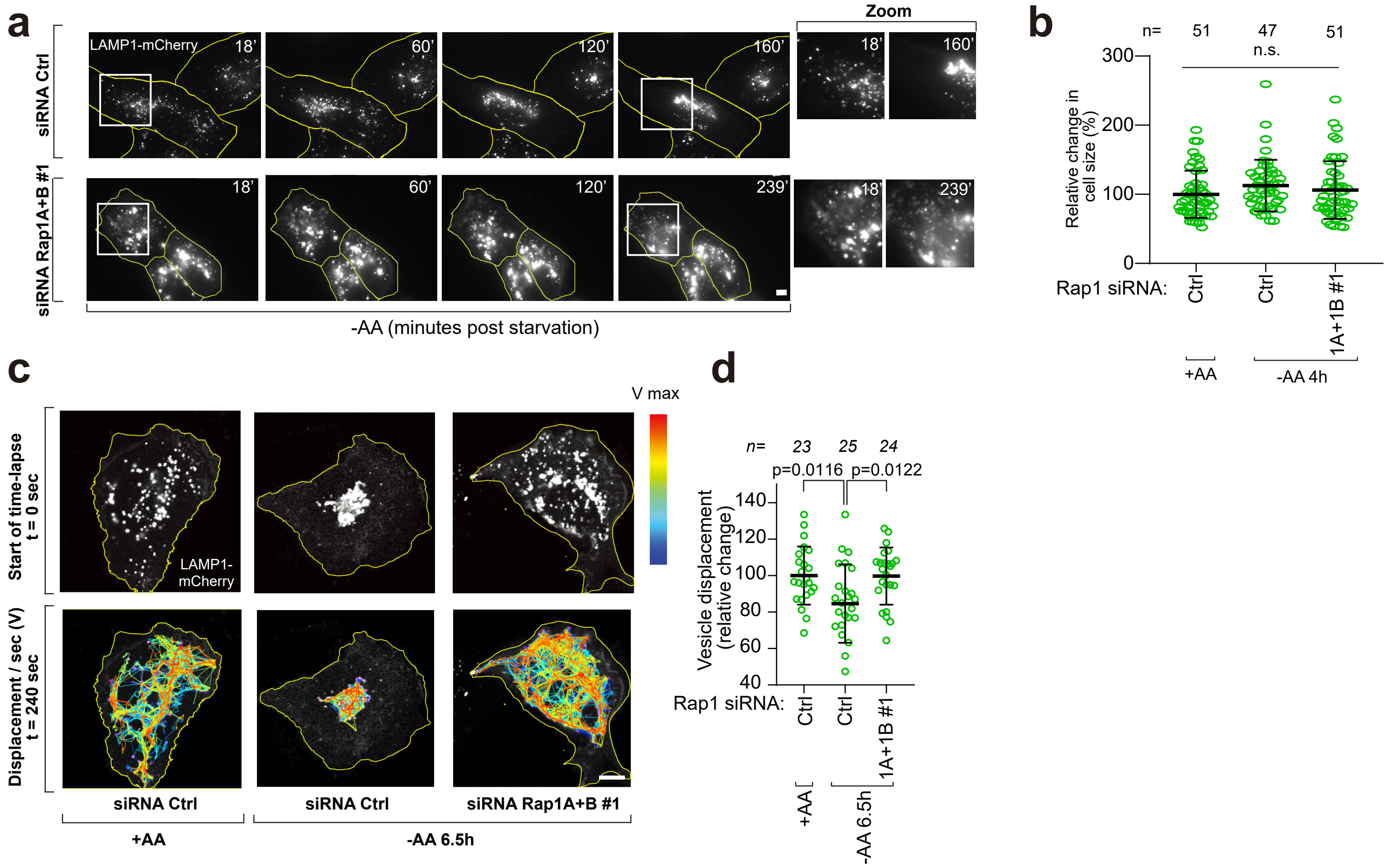

Supplementary Figure 7

Supplementary Figure 7. Analysis of the distribution of lysosomes in live cells upon amino acid starvation

(a) U2OS-Lamp1-mCherry expressing cells were transfected with siRNA against Rap1A+B and chased every minute after 15 minutes of complete amino acid starvation for up to 4 hours, using time-laps fluorescence microscopy. Representative images from Supplementary Movies 1 and 3 are shown at the indicated time (minutes) of starvation. Experiments were repeated at least three times. **(b)** The relative change in cell size to control cells was quantified from multiple confocal images of amino acid starved Rap1A+B depleted U2OS cells. **(c-d)** Distribution and dynamics of LAMP1-mCherry vesicles in Rap1A+B-depleted U2OS cells deprived of amino acids. Representative images at the start (t=0) and end (t=240 sec) of a time lapse analysis, with vesicle displacement rates displayed in the lower panel (blue: low mobility, red: max mobility). The relative change in vesicle displacement is quantified in (**d**). Also see Supplementary Movies 4-6. Scalebars: **(a,c)** 10 μm., *n* denotes the number of individual cells analyzed across 3 independent experiments and data are presented as mean values +/- s.d. n.s.= not significant (P>0.05); one-way ANOVA with Tukey's post hoc test**.** In **b**, p=0.2322 and 0.6938 between Ctrl vs Ctrl-AA, and Ctrl vs siRap1-AA, respectively. The microscopic fields imaged were randomly selected. See Source Data File for statistics source data.

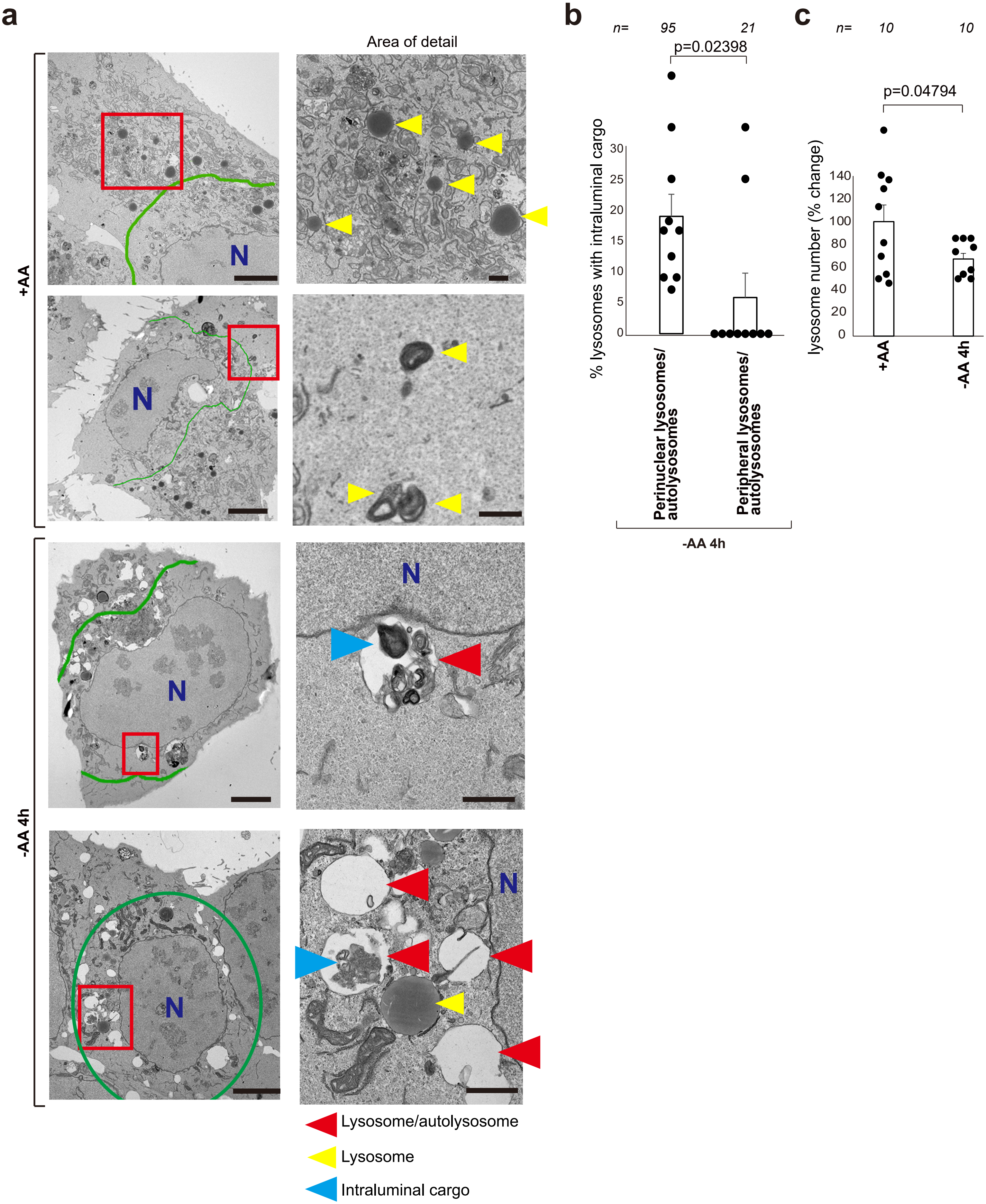

Supplementary Figure 8

Supplementary Figure 8. Analysis of the ultrastructure of the perinuclear region of amino acid starved cells

(a-c) U2OS cells were subjected to four hours of amino acid starvation and analyzed by electron microscopy. Representative images are shown in **(a)**. Experiment was repeated two times. An increase in cleared lysosome/autolysosome-like structures was observed in amino acid starved cells. The fraction of lysosomes/autolysosomes in which intraluminal cargo could be observed was quantified for perinuclear (<5 μm from the nucleus) and peripheral (>5 μm from the nucleus) lysosomes/autolysosomes across two independent experiments **(b)**. The perinuclear boundary is depicted in green. *n* denotes the number of lysosomes assessed. In **(c)**, the change in total number of lysosomes, which include cleared and electron dense structures, is quantified for amino acid starved cells across two independent experiments. *n* denotes the number of cells analyzed.

Yellow arrowheads indicate lysosomes, blue arrowheads indicate intraluminal cargo and red arrowheads cleared lysosomes/autolysosomes. N=nuclei. Scale bars, left panels: 5 μm, right panels: 1 μm. Student's *t*-test; two-sided, unpaired**.** Data are presented as mean values +/- s.e.m. See Source Data File for statistics source data.

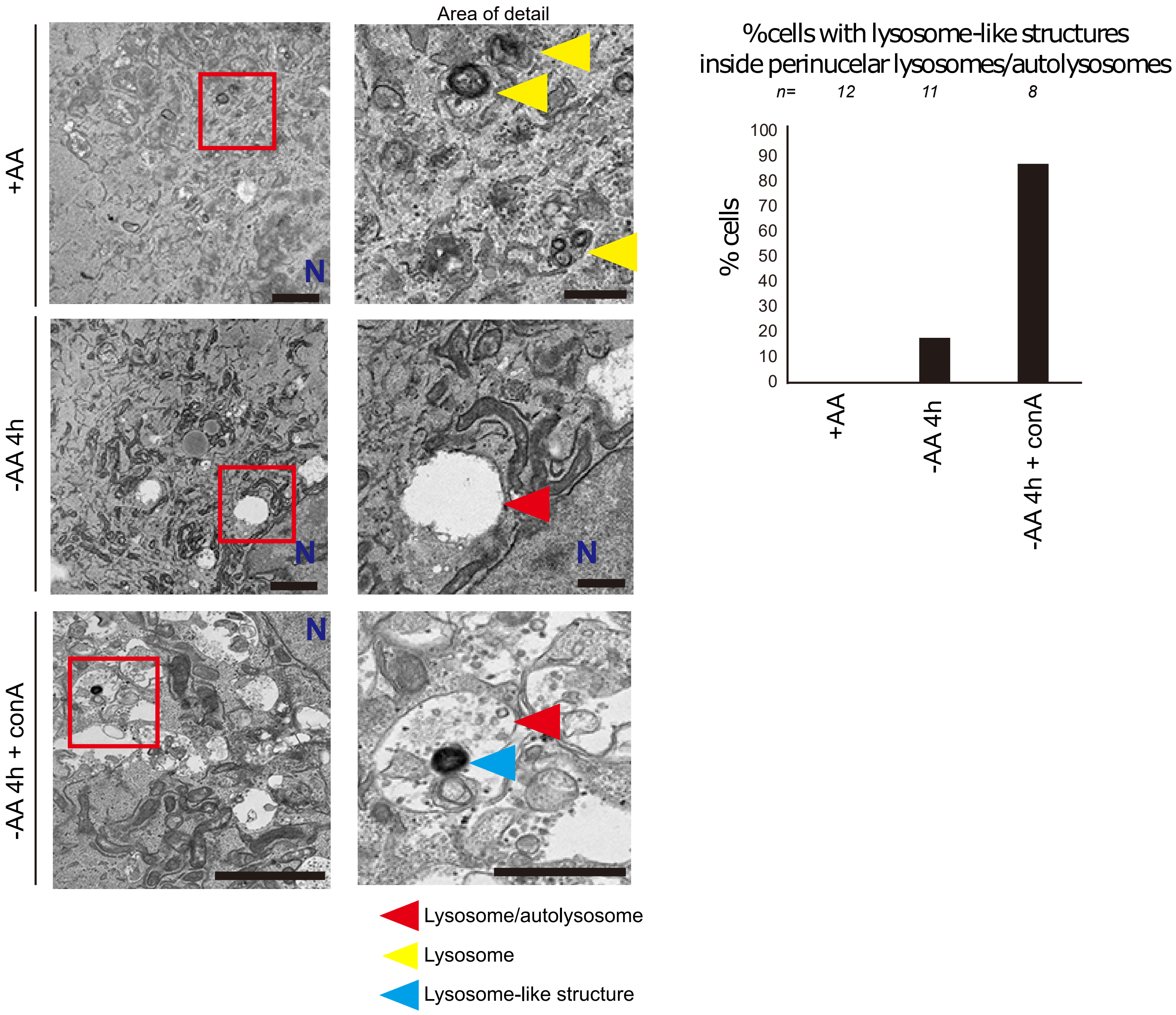

Supplementary Figure 9

Supplementary Figure 9. Analysis of the ultrastructure of the perinuclear region of amino acid starved cells where lysosome degradative function is blocked

Representative transmission electron microscopy images of U2OS cells subjected to four hours of amino acid starvation, under which lysosome degradative function was blocked by 100 nM conA. An increase in cleared lysosomes/autolysosomes with intraluminal lysosome-like structures was observed in the perinuclear region (<5 μm from the nucleus) when lysosome-degradation was blocked. The fraction of cells in which at least one electron dense lysosome structure was observed inside a lysosome/autolysosome is quantified to the right (+AA: no structures observed in 12 cells, -AA 4h: structures observed in 2 out of 11 cells=18.18%, –AA + ConA: structures observed in 7 of 8 cells=87.5%). Cells were pretreated with conA for one hour before treatment was initiated. Yellow arrowheads indicate lysosomes, blue arrowheads indicate lysosome-like structures and red arrowheads cleared lysosomes/autolysosomes. N=nuclei. *n*=number of cells analyzed. Scale bars, left panels: 2.5 μm, right panels: 1 μm. See Source Data File for statistics source data.

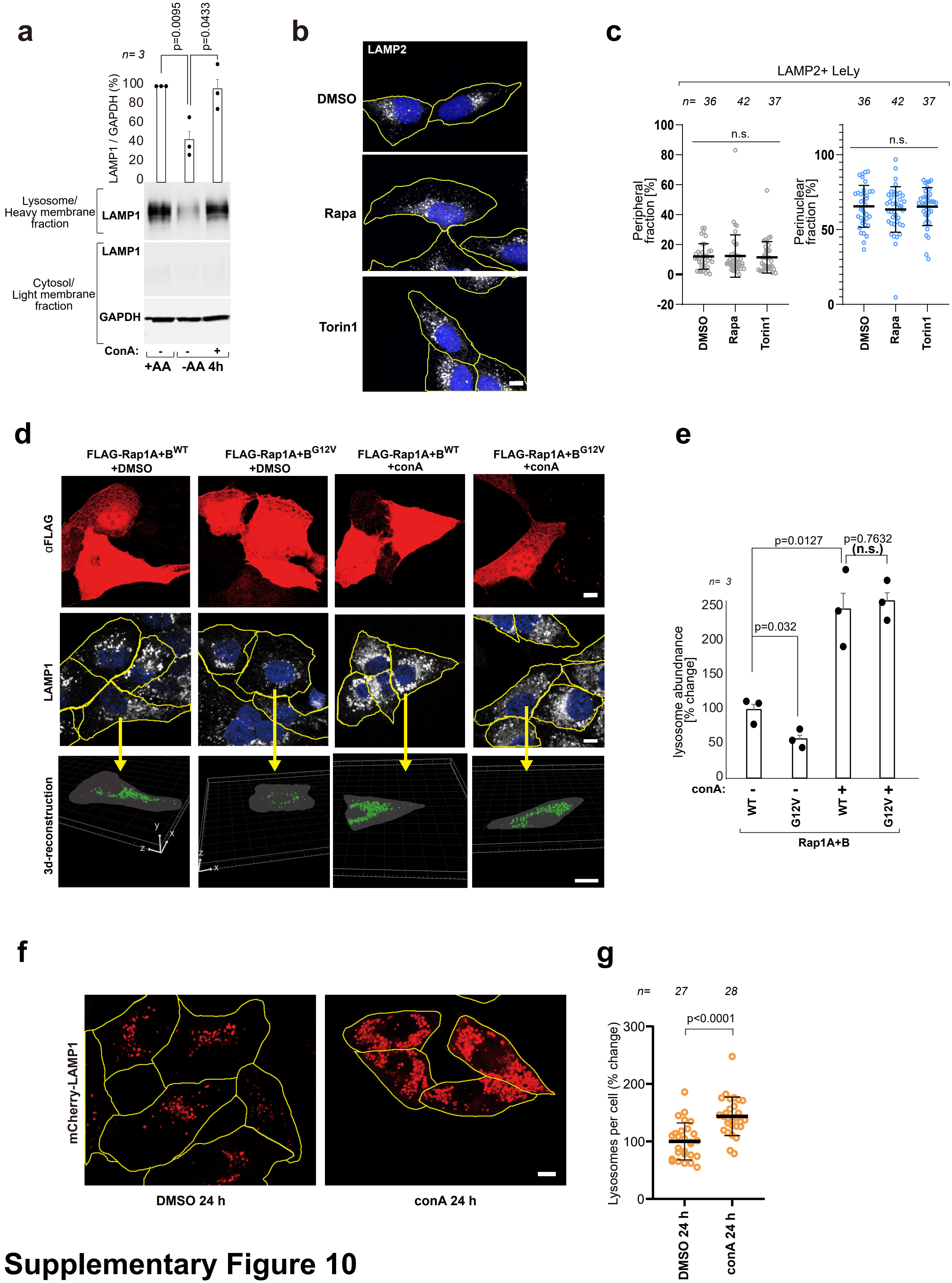

Supplementary Figure 10

Supplementary Figure 10. Lysosome degradative function is required for Rap1 suppression of lysosome abundance

(a) U2OS cells were subjected to amino acid starvation in the presence or absence of 100 nM conA for four hours, whereafter lysates were separated into lysosome-enriched heavy membranes and cytosolic/light membranes fractions, and analyzed by immunoblotting for the endogenous amounts of the indicated proteins. Cells were pretreated with conA for one hour before treatment was initiated.

(**b-c**) Representative z-stack projections of the endogenous LAMP2+ lysosome (LeLy) staining in U2OS cells treated for four hours with DMSO control, 20 nM Rapamycin or 50 nM Torin1. Peripheral and perinuclear lysosomal fractions are quantified in **(c)**. Experiment was repeated at least three times. Scale bar: 10 μm

(d) Representative Z-stack projections and 3d-reconstructions of the change in lysosomal abundance in U2OS cells transfected with the indicated FLAG-Rap1 cDNAs after treatment with 100 nM conA for 24 hours. 3d-reconstruction analysis was performed on all FLAG-Rap1 cDNA expressing cells in the field of view. The yellow arrow indicates one representative cell for which the 3d-reconstruction is shown below. The percentage change in lysosomal abundance is quantified in **(e)**. The number of cells analyzed to quantify lysosome abundance is shown in Supplementary Fig 14. Scale bars: upper and middle panel: 10 μm, lower panel: 20 μm.

(f-g) Representative images of U2OS mcherry-Lamp1 expressing cells treated with conA for 24 hours. The relative change in lysosome numbers normalized to the total cell area is quantified in **(g)** for *n* individual cells across three independent experiments. For details, refer to Materials and Methods. Scale bar: 10 μm

Cell boundaries are depicted in yellow. Cell nuclei (DAPI) is displayed in blue. The microscopic fields imaged were randomly selected. In **(c,g)**, *n* denotes the number of individual cells analyzed across 3 independent experiments and data are presented as mean values +/- s.d. In **(a,e)**, *n* denotes the number of individual experiments and data are presented as mean values +/- s.e.m. The number of cells analyzed to quantify lysosome abundance is shown in Supplementary Figure 14. n.s.= not significant (P>0.05); Student's *t*-test; two-sided, unpaired (**a,e,g**), one-way ANOVA with Tukey's post hoc test **(c).** In **(c)**, p=0.9952 and 0.9753 for DMSO vs Rapa and DMSO vs Torin1, peripheral distribution, and p=0.7966 and 0.9982 for DMSO vs Rapa and DMSO vs Torin1, perinuclear distribution, respectively. See Source Data File for statistics source data. Uncropped images of blots are shown in Supplementary Figure 21

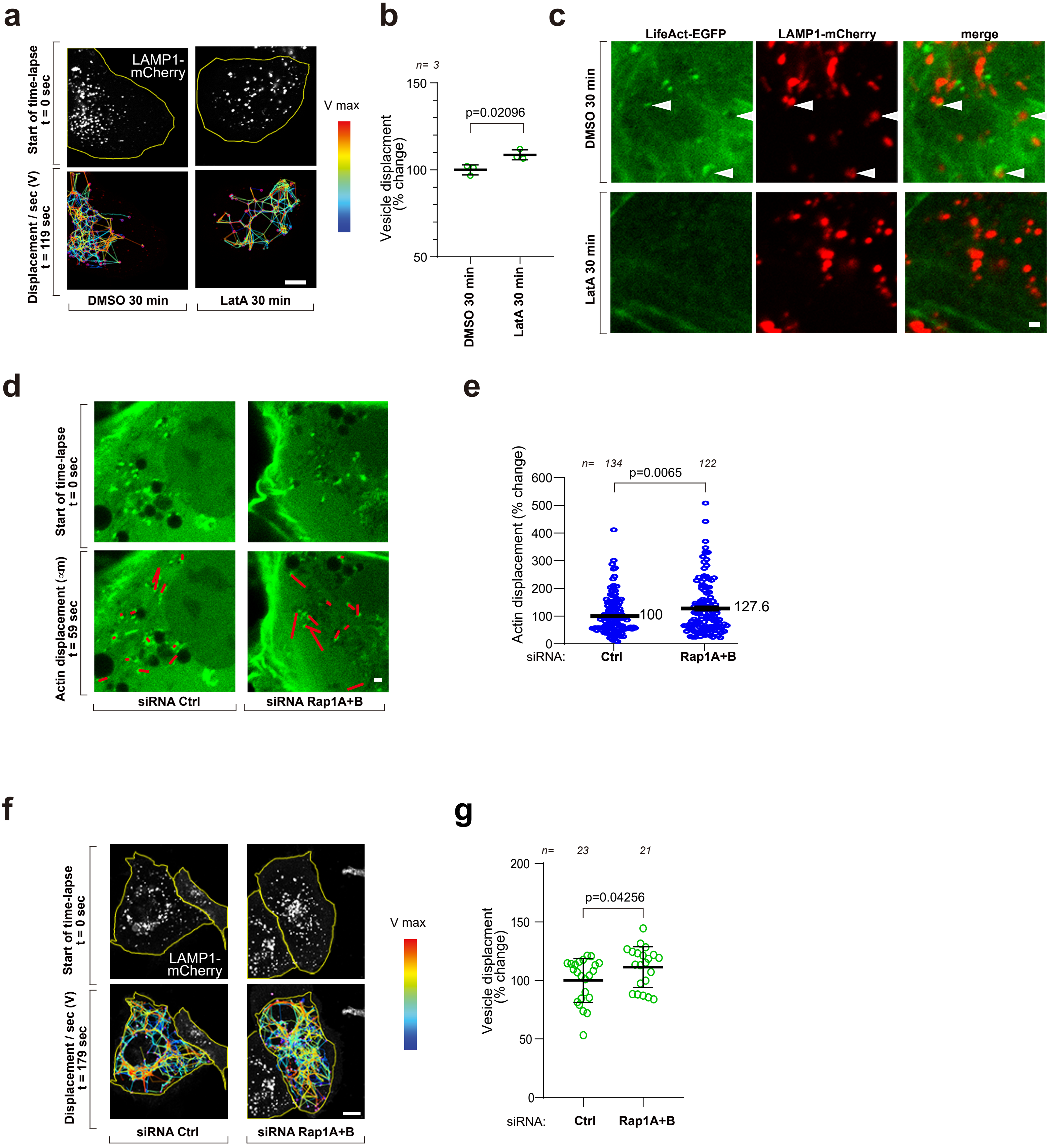

Supplementary Figure 11

Supplementary Figure 11. Lysosome-associated actin is coordinated by Rap1

(a-b) Distribution and dynamics of LAMP1-mCherry vesicles in U2OS cells treated with 0.1 μM latA for 30 minutes. Representative images at the start (t=0) and end (t=119 sec) of a time lapse analysis, with vesicle displacement rates displayed in the lower panel (blue: low mobility, red: max mobility). The relative change in vesicle displacement is quantified in **(b)** for three independent experiments analyzing a minimum of 12 individual cells. Also see Supplementary Movies 7-8. Scale bar: 10 μm. *n* denotes the number of individual experiments and data are presented as mean values +/- s.d.

(c) Representative images at the start of a time lapse of LifeAct-EGFP expressing U2OS-Lamp1-mcherry cells treated with 0.1 μM LatA for 30 minutes. In DMSO treated cells, cytoplasmic F-actin patches dynamically interact with lysosomes, as indicated by white arrowheads. Also, see Supplementary Movies 9-10. Experiment was repeat at least three times. Scale bar: 1 μm.

(d-e) The change in displacement of LifeAct-EGFP labelled actin patches upon Rap1A+B depletion was assessed in HEK293A cells. Representative images at the start (t=0) and end (t=59 sec) of a time lapse analysis, with the change in actin displacement quantified in **(e).** *n* denotes the number of actin patches analyzed across three individual experiments, analyzing at least 10 cells, and data are presented as mean values +/- s.d. The scorer was blinded to treatment. Also, see Supplementary Movies 11-12. Scale bar: 1 μm.

(f-g) Distribution and dynamics of LAMP1-mCherry vesicles in Rap1A+B depleted U2OS cells. Representative images at the start (t=0) and end (t=179 sec) of a time lapse analysis, with vesicle displacement rates displayed in the lower panel (blue: low mobility, red: max mobility). The relative change in vesicle displacement is quantified in **(g)**. *n* denotes the number of individual cells and data are presented as mean values +/- s.d. Scale bar: 10 μm. Also see Supplementary Movies 13-14. Cell boundaries are depicted in yellow. Cell nuclei (DAPI) is displayed in blue. The microscopic fields imaged were randomly selected. Experiments were repeated at least three times. Student's *t*-test; two-sided, unpaired. See Source Data File for statistics source data.

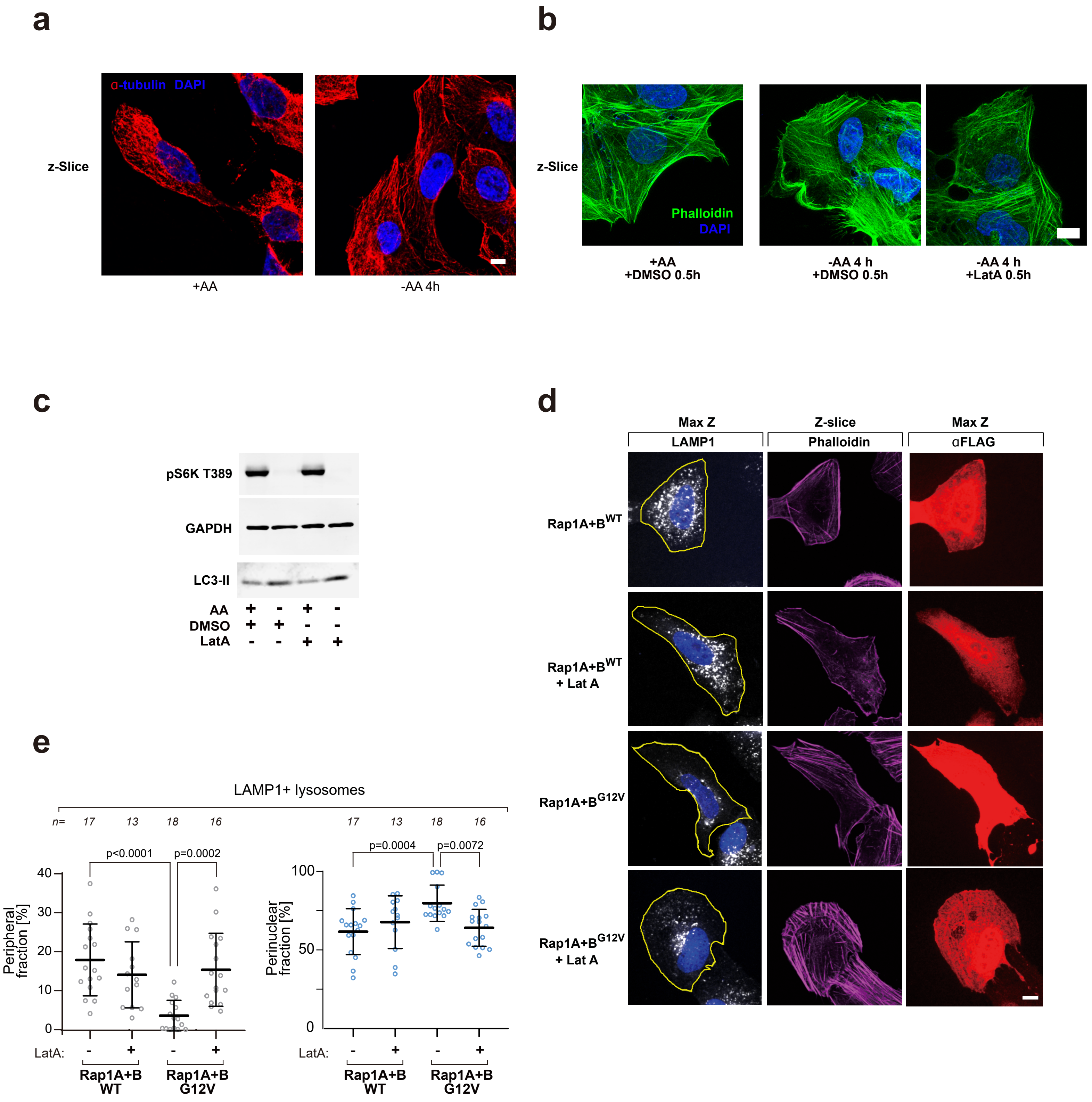

Supplementary Figure 12

Supplementary Figure 12. Rap1 coordinates lysosomes through the regulation of actin

(a) Representative images are shown from a single confocal plane of α-tubulin stained U2OS cells subjected to amino acid starvation.

(**b-c**) U2OS cells that had been amino acid starved for 3.5h and treated with either DMSO or 0.1 μM Lat A for an additional 30 minutes (total 4 hours of starvation). In **(b)**, representative images from a single confocal plane are shown of Phalloidin stained cells. In **(c)** mTORC1 and autophagy associated signaling was assessed by immunoblotting endogenous proteins, as indicated.

In **(d-e)**, lysosomal distribution was assessed in U2OS cells that had been transfected with the indicated FLAG-Rap1A+B cDNAs and treated with either DMSO or 0.1 μM latrunculin A (Lat A) for two hours. Quantifications are shown in **(e)**. *n* denotes the number of individual cells analyzed across 3 independent experiments and data are presented as mean values +/- s.d; one-way ANOVA with Tukey's post hoc test. All experiments were repeated at least three times. In **(e)**, p=0.4919 for Rap1 Ctrl Vs Rap1 LatA, peripheral fraction, and p=0.4695 for Rap1 Ctrl Vs Rap1 LatA, perinuclear fraction. Scale bars: 10 μm. The microscopic fields imaged were randomly selected. See Source Data File for statistics source data.

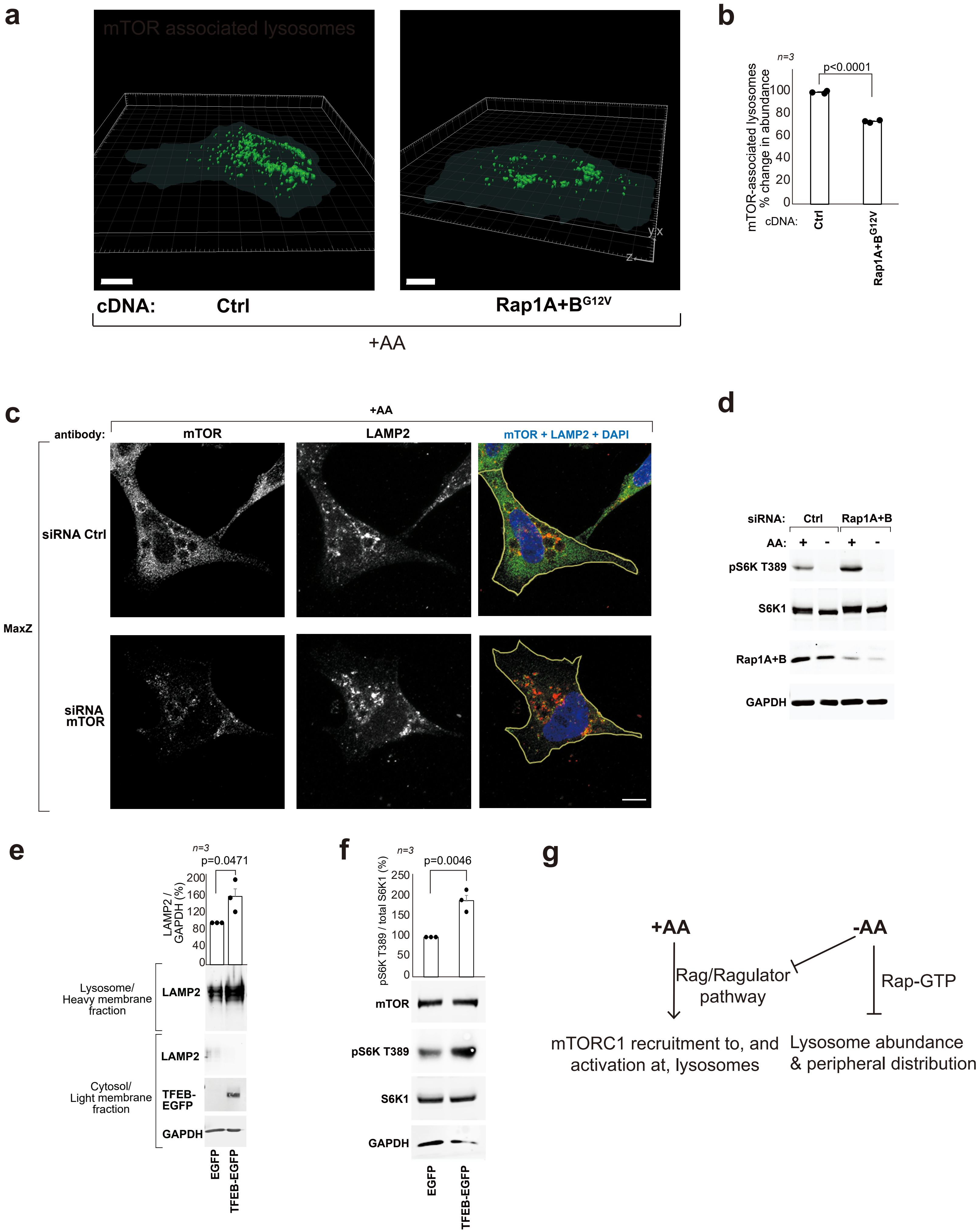

Supplementary Figure 13

Supplementary Figure 13. Analysis of mTOR distribution and signaling

(a-b) Representative 3D-reconstructions of mTOR associated lysosomes from HEK293A cells expressing the indicated Rap1A+B cDNA (**a**), with quantification shown in (**b**) for three individual experiments. The number of cells analyzed in the quantification of lysosome abundance across three individual experiments is shown in Supplementary Figure 14.
(c) mTOR was targeted with two siRNAs and subjected to immunocytochemistry analysis for mTOR (green) and LAMP2 (red). Cell boundaries are depicted in yellow and cell nuclei (DAPI) are shown in blue. Scalebar:10 μm. Experiment was repeated at least three times.
(d-f) Immunoblots showing S6K1 phosphorylation **(d,f)** and LAMP2 levels **(e)** in Rap1A+B siRNA-depleted **(d)** or TFEB-EGFP expressing cells **(e-f)**. HEK293A cells were maintained in full growth conditions **(e-f)** or subjected to 1 hour of complete amino acid starvation **(d)**, as indicated. In **(e)**, cell lysates were separated into lysosome-enriched heavy membranes and cytosolic/light membranes fractions. Experiments were repeated at least three times.
(g) Model: Rap1 suppresses lysosome abundance and peripheral expansion in response to limitations in amino acids (right). When amino acid levels are sufficient, mTORC1 is recruited to and activated on lysosomes by Rag-GTPases (left). Also, see model in Fig 5f.

Scalebar:10 μm. Cell boundaries are depicted in yellow. Cell nuclei (DAPI) is displayed in blue. The microscopic fields imaged were randomly selected. *n* denotes the number of individual experiments and data are presented as mean values +/- s.e.m. Student's *t*-test; two-sided, unpaired. pS6K T389 and total S6K1 were processed on separate blots due to technical reasons. See Source Data File for statistics source data. Uncropped images of blots are shown in Supplementary Figure 21.

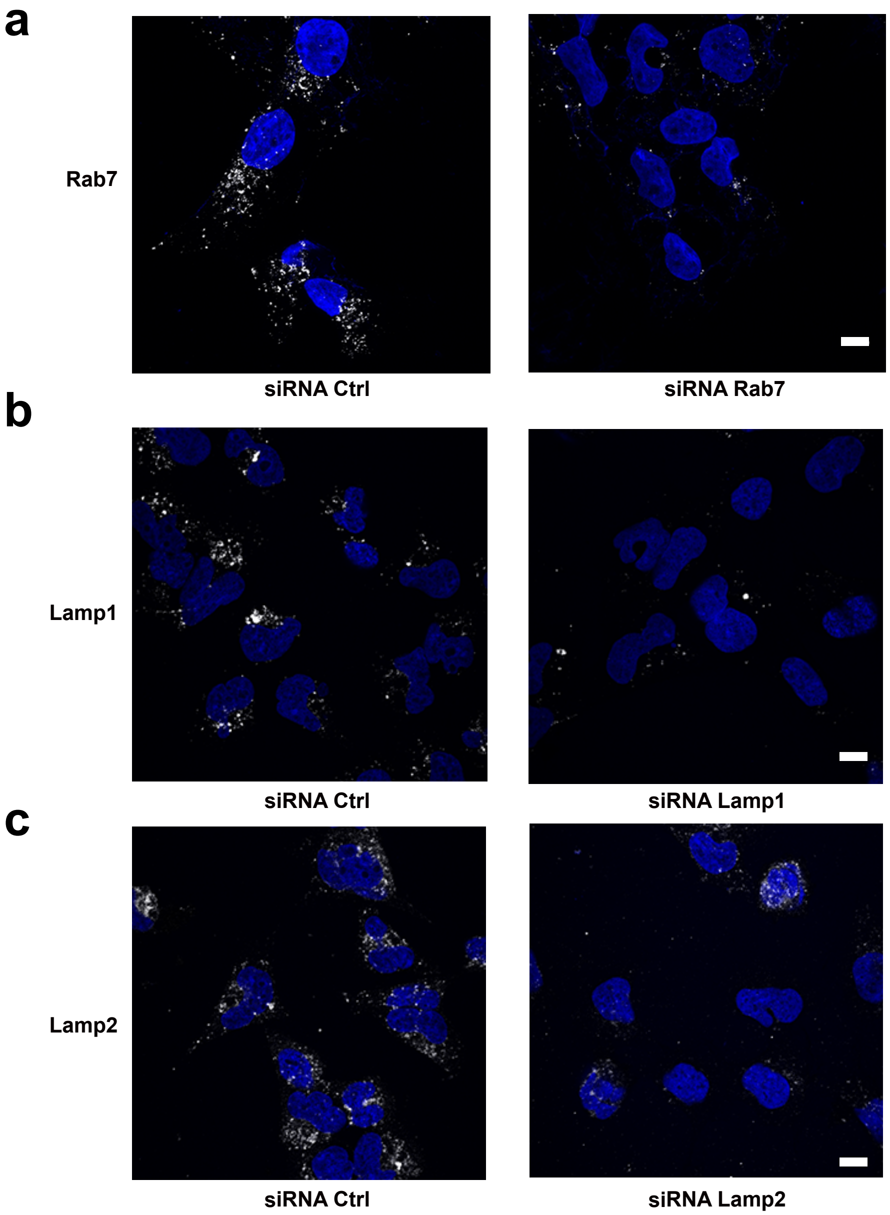

d

**n= number of cells analyzed across atleast three individual experiments**						
**Fig 3e-f**	ctrl n=22	wt n=21	G12V n=22			
**Fig3 i-j**	siCtrl +AA n=26	siCtrl -AA n=24	siRap1A+B #1 -AA n=23	siRap1A+B #2 -AA n=25	siRap1A+B #1 +AA n=27	siRap1A+B #2 +AA n=25
**Sup Fig 4a-b**	siCtrl +AA n=30	siRap1A+B #1 +AA n=24				
**Fig5 d**	siCtrl +AA n=31	siRap1A+B #1 +AA n=27				
**Sup Fig 13a**	Ctrl n=15	Rap1A+B G12V n=17				
**Sup Fig 4f-g**	siCtrl + EV n=77	siRap1A+B + EV n=94	siRap1A+B + wild-type Rap1A+B n=49			
**Sup Fig 6b-c**	EV +AA n=57	EV -AA n=38	dnATG4 C74A +AA n=21	dnATG4 C74A -AA n=20		
**Fig4b-c SupFig10d-e**	WT DMSO n=26	G12V DMSO n=23	WT CQ n=23	G12V CQ n=24	WT conA n=19	G12v conA n=21

Supplementary Figure 14

Supplementary Figure 14.

(a-c) HEK293A cells were transfected with the indicated siRNAs and immunostained for Rab7 **(a)**, Lamp1 **(b)** or Lamp2 **(c)**. Experiments were repeated at least three times.

(d) For 3d-reconstruction analyzes, the number of individual cells (*n*) analyzed across at least three individual experiments are specified for the indicated experiments. 3D-reconstructions were made from confocal images of LAMP1 or LAMP2 and mTOR coimmunostained cells. The microscopic fields imaged were randomly selected. Scalebar:10 μm.

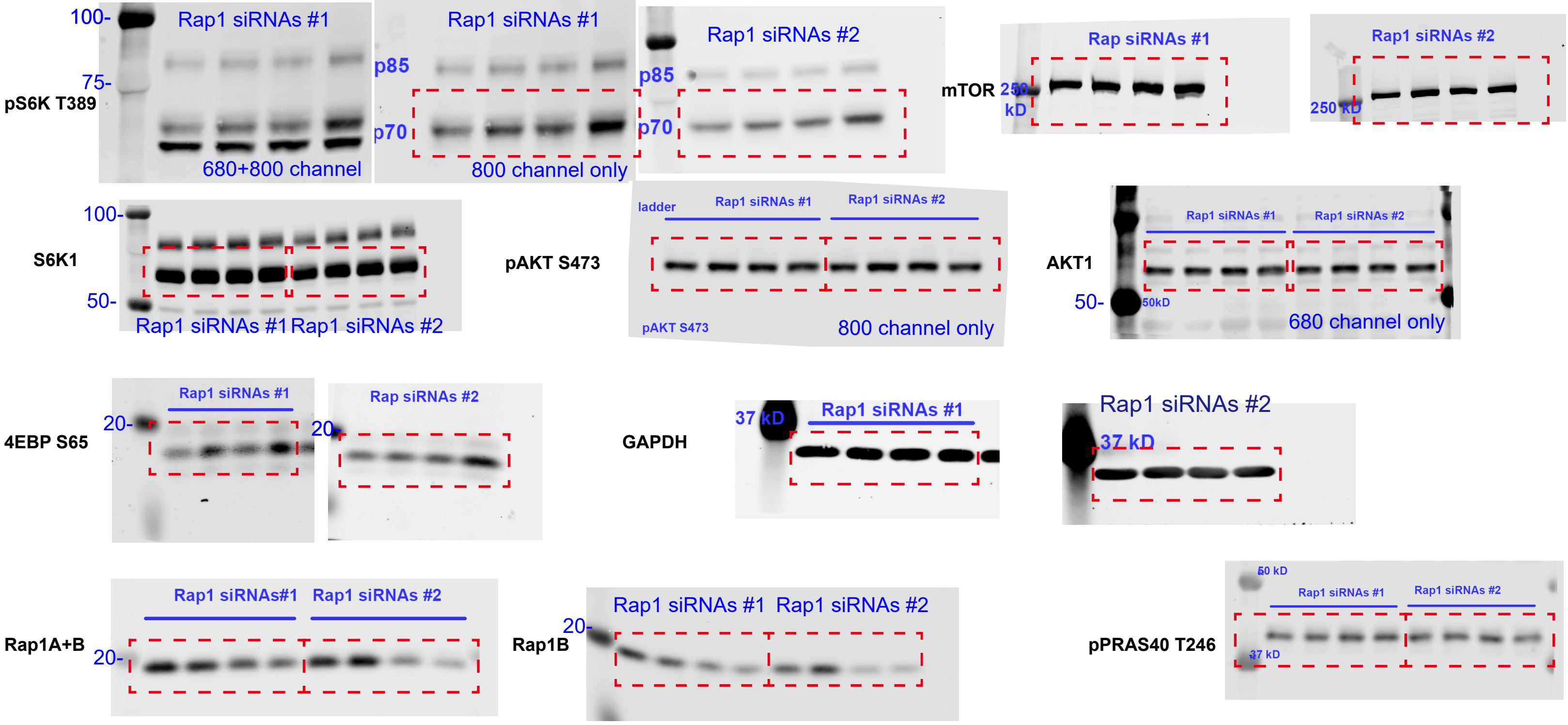

Figure 1a

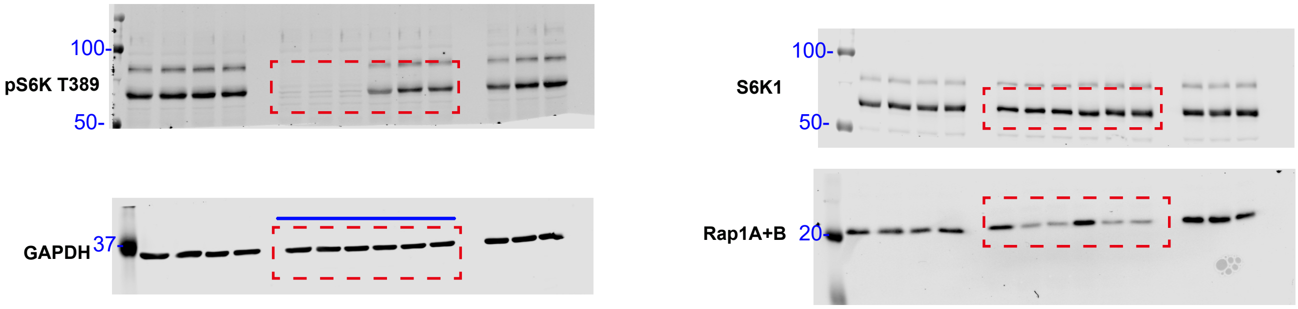

Figure 1b

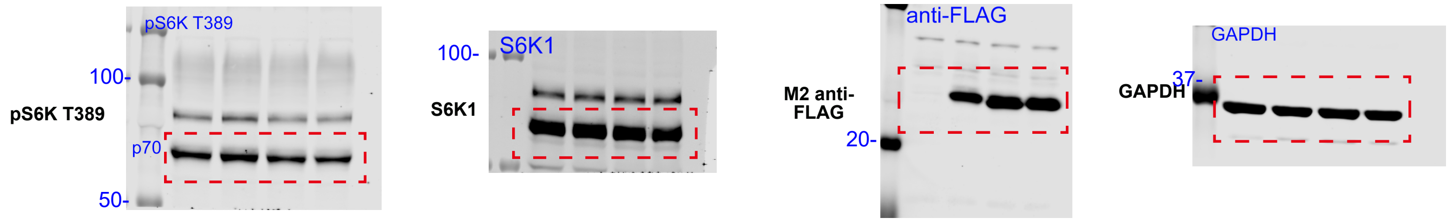

Figure 1c

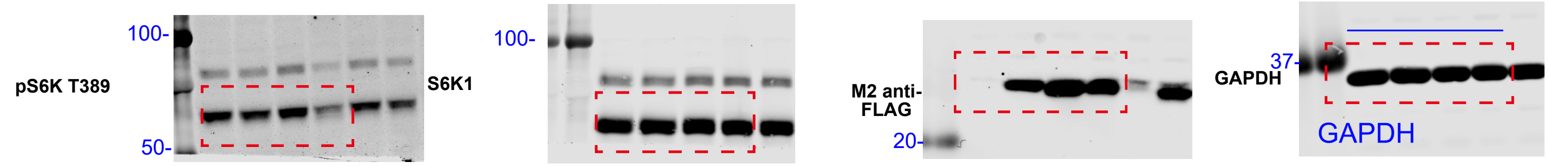

Figure 1d

Supplementary Figure 15

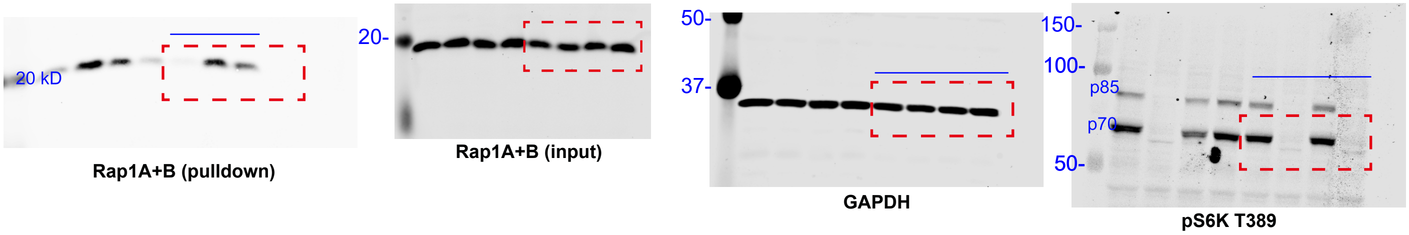

Figure 2a

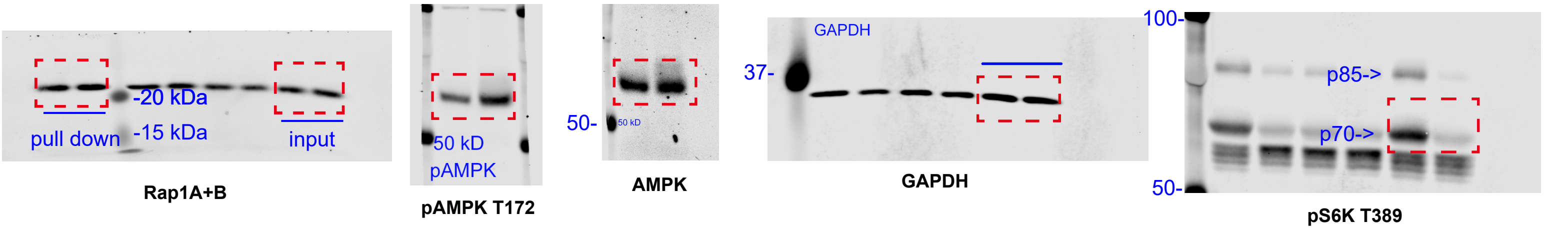

Figure 2b

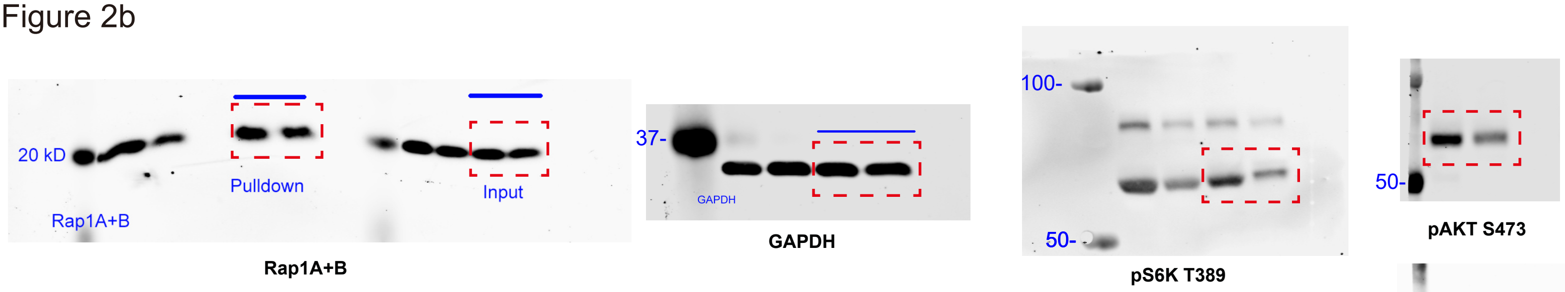

Figure 2c

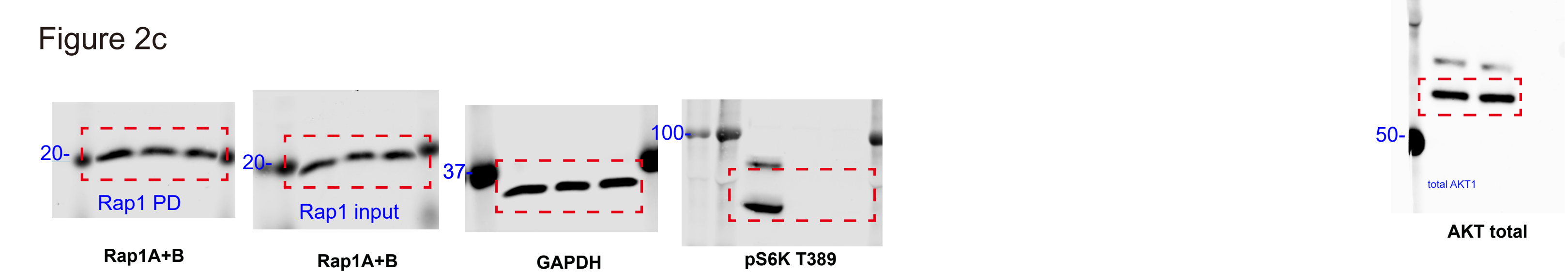

Figure 2d

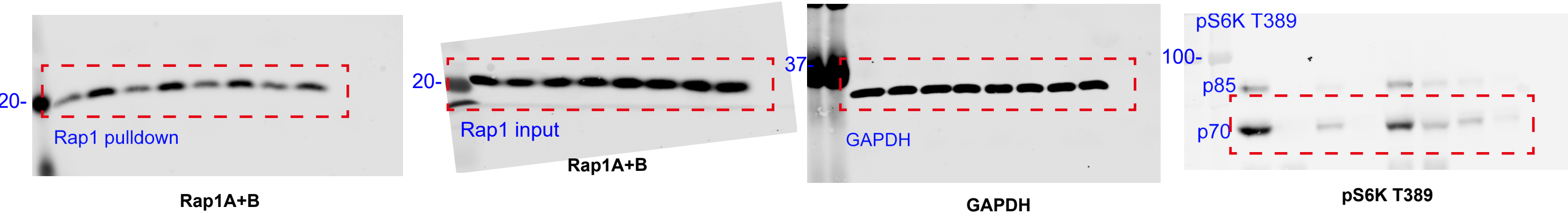

Figure 2e

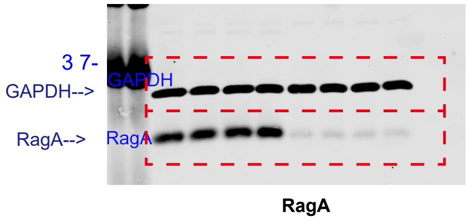

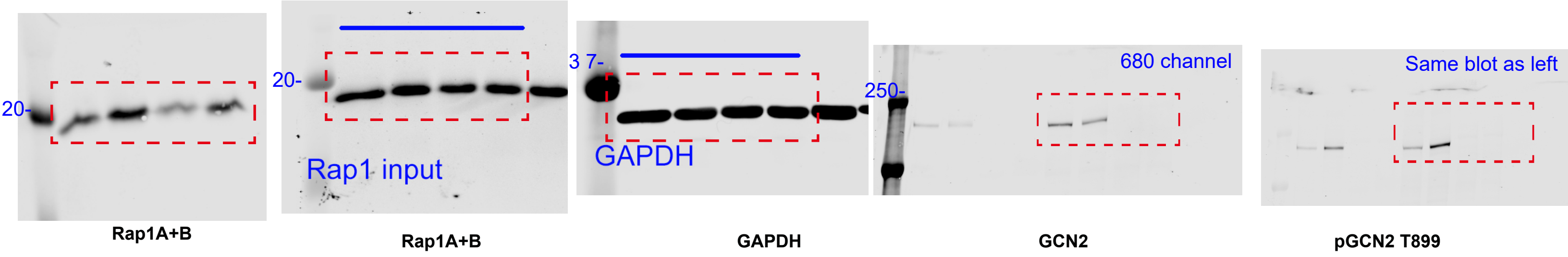

Figure 2f

Supplementary Figure 16

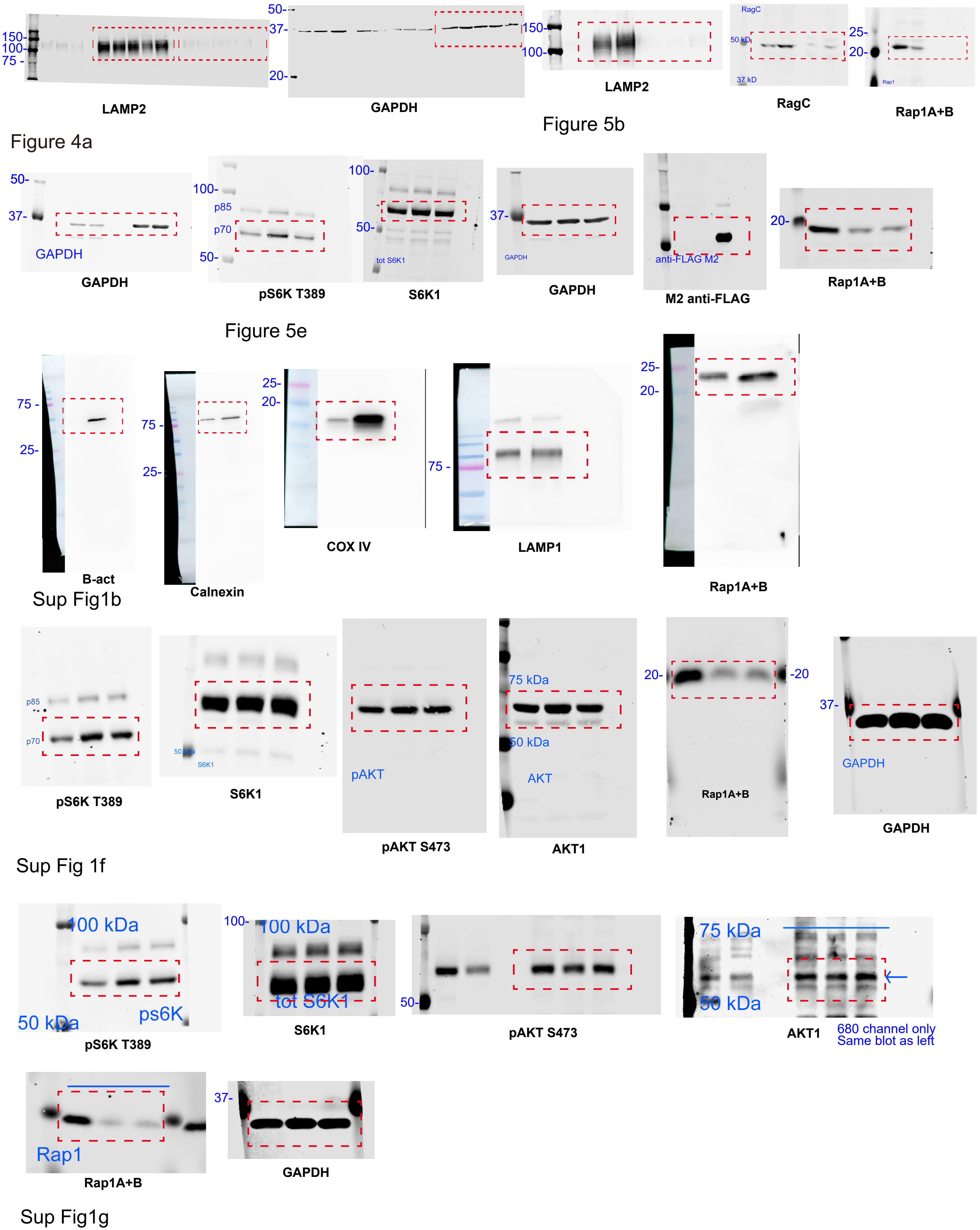

Supplementary Figure 17

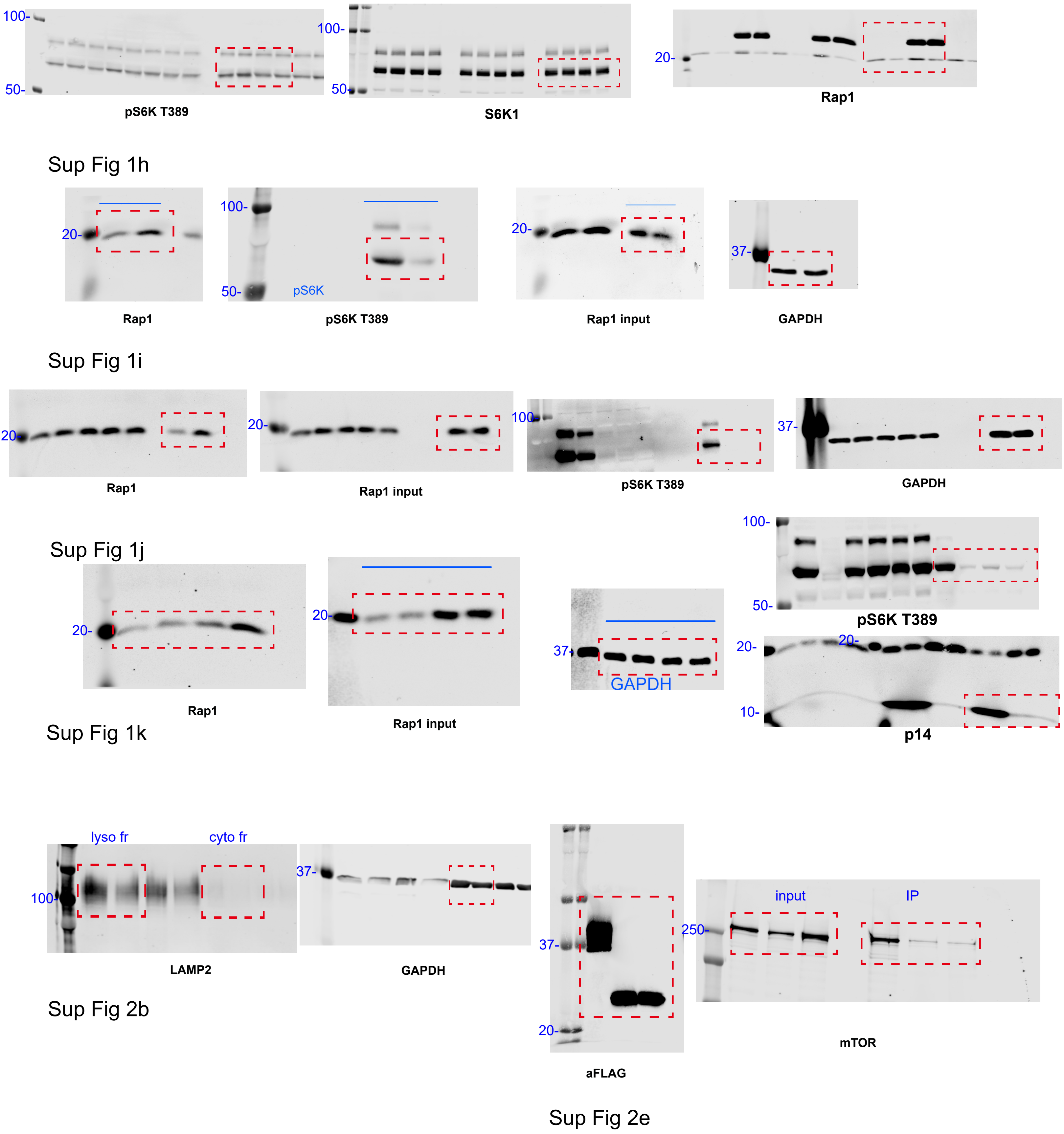

Supplementary Figure 18

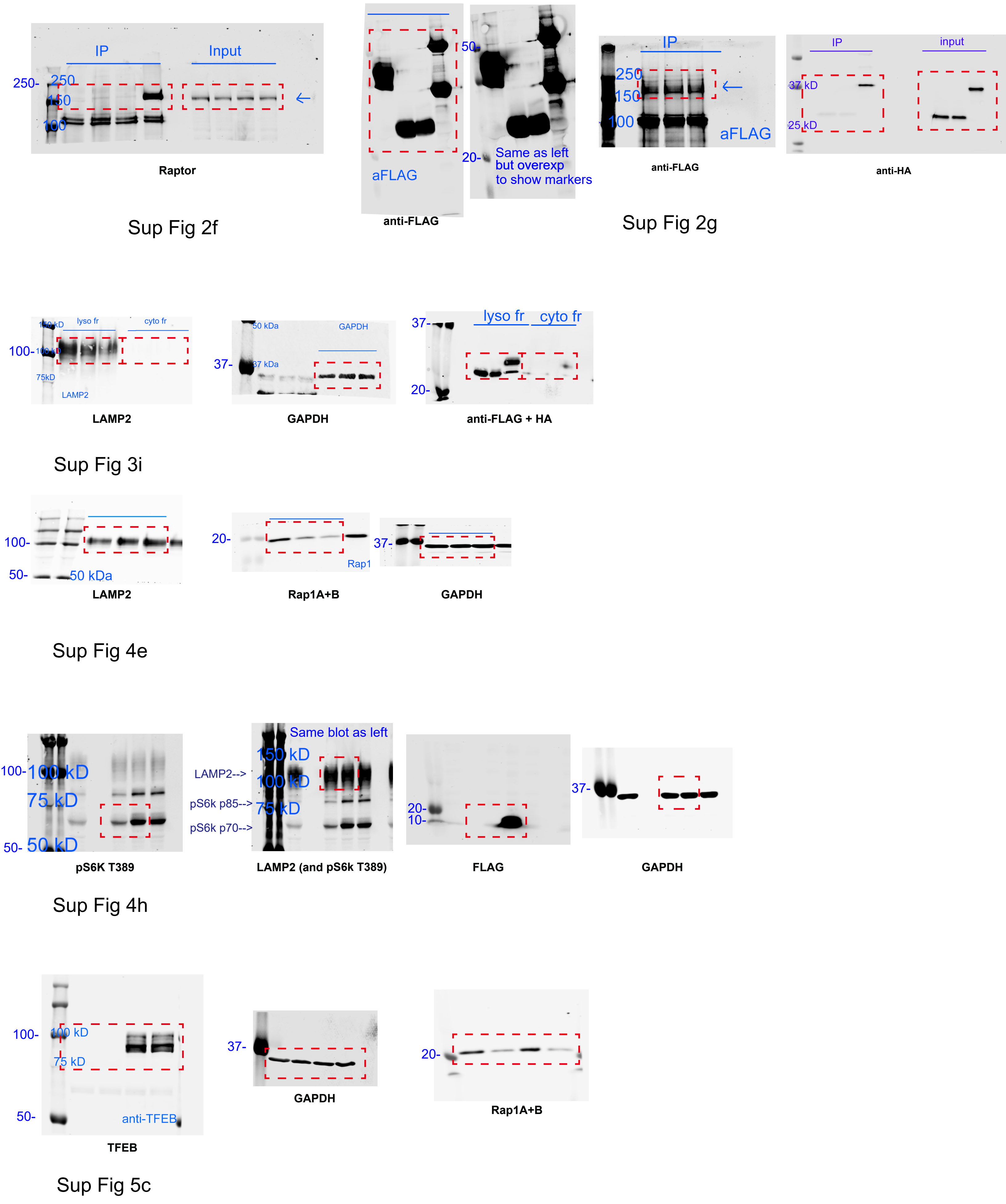

Supplementary Figure 19

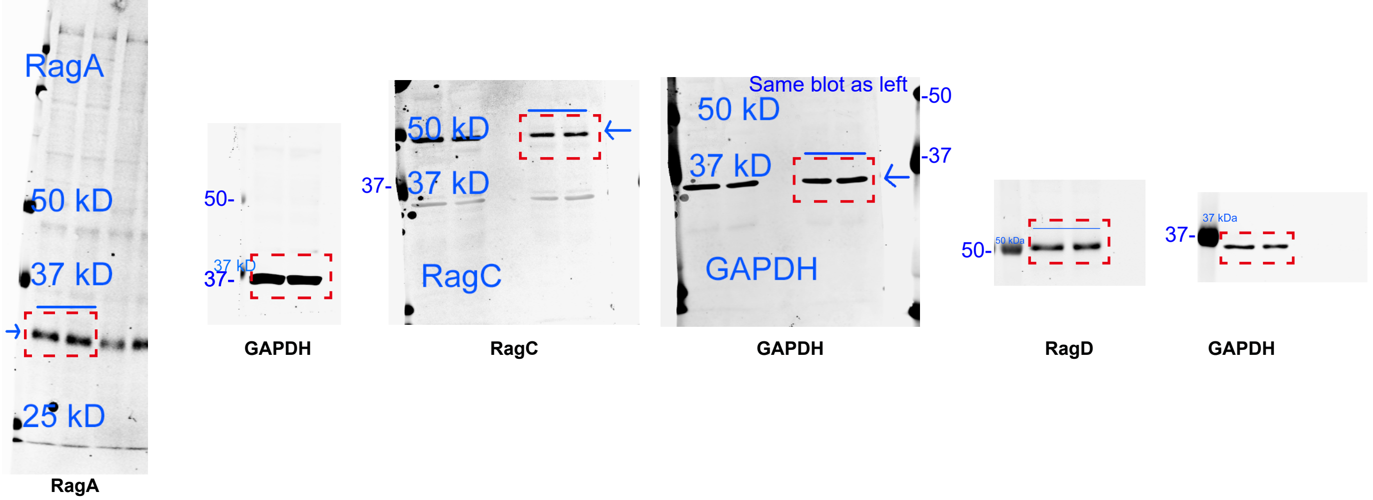

Sup Fig 5e

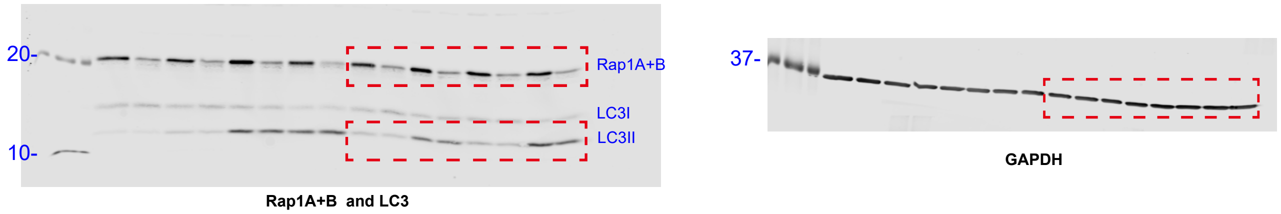

Sup Fig 5f

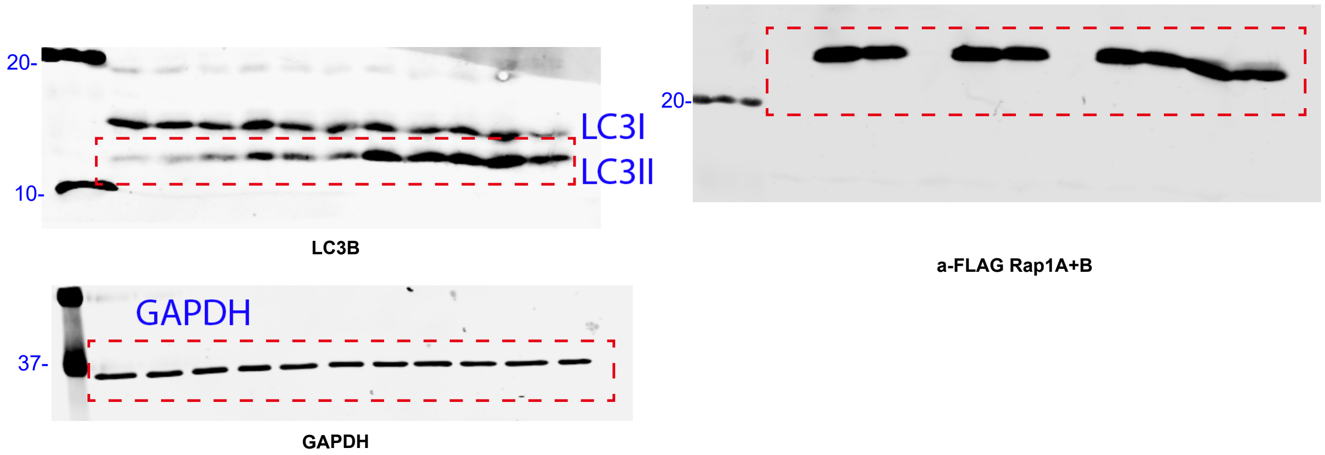

Sup Fig 5g

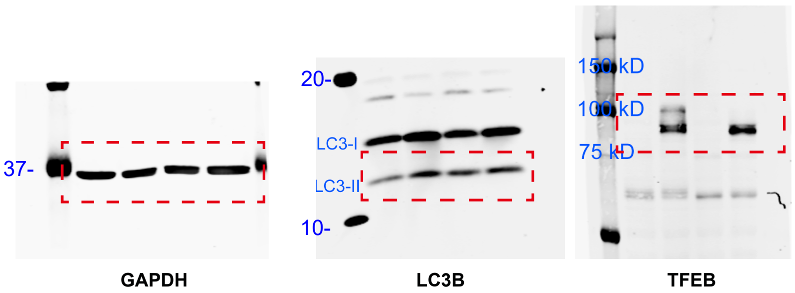

Sup Fig 5h

Supplementary Figure 20

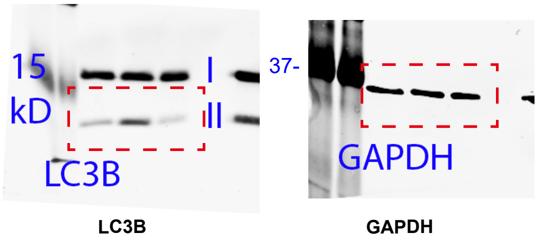

Sup Fig 6d

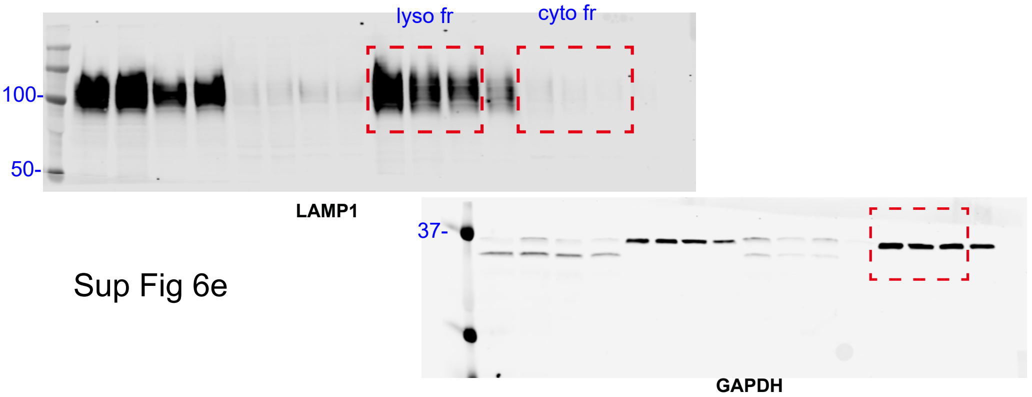

Sup Fig 6e

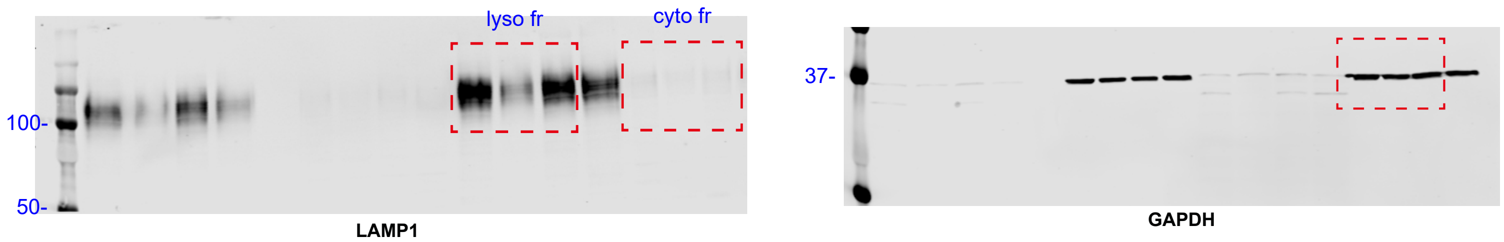

Sup Fig 10a

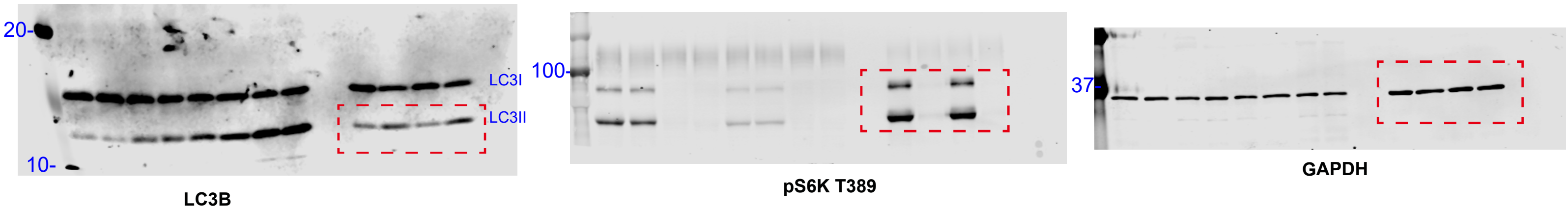

Sup Fig 12c

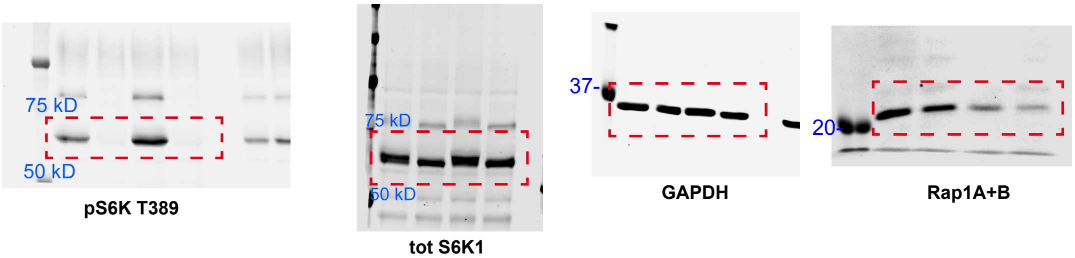

Sup Fig 13d

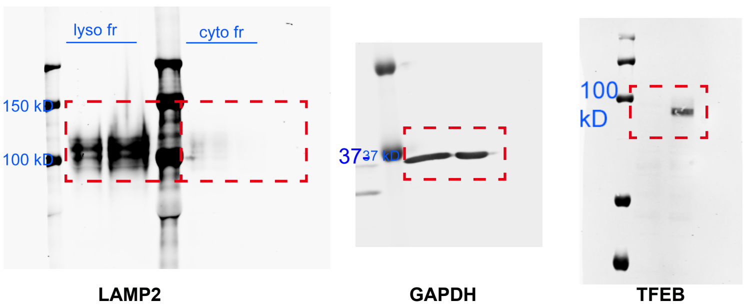

Sup Fig 13e

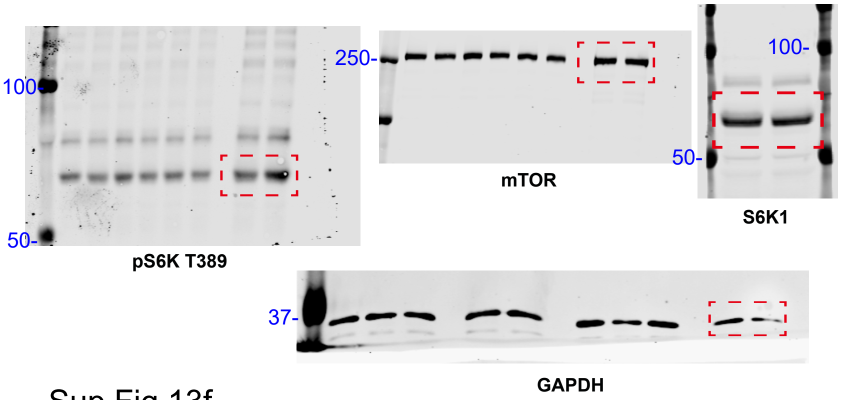

Sup Fig 13f

Supplementary Figure 21

Supplementary Figure 15-21. Uncropped immunoblots.

Uncropped immunoblots are shown for the indicated figures. Molecular weight markers were captured in the Odyssey 680 channel. In Fig 1a (pS6K T389 and pAKT and AKT), Fig 2f (pGCN2 and GCN2), Sup Fig 4h (pS6K and LAMP2), two antibodies were captured simultaneously and the marker is shown in the 680 channel.

Supplementary References

1. J. Riedl *et al.*, Lifeact: a versatile marker to visualize F-actin. *Nat. Methods*. **5**, 605–7 (2008).
