# Supplementary material for: Rap1-GTPases control mTORC1 activity by coordinating lysosome organization with amino acid availability
Source: Nat Commun. 2020 Mar 17;11:1416. doi: 10.1038/s41467-020-15156-5 (PMC7078236; doi:10.1038/s41467-020-15156-5)
Supplement: Supplementary file 2 — Description of Additional Supplementary Files [file 41467_2020_15156_MOESM2_ESM.pdf]

Description of Additional Supplementary Files

File Name: Supplementary Movie 1

Description: U2OS-Lamp1-mCherry expressing cells were transfected with siRNA control and chased every minute after 10 minutes of complete amino acid starvation for up to 217 minutes, using time-lapse fluorescence microscopy. 10 μm scale bar is shown in the first frame. Numbers indicate the time (minutes) of amino acid starvation. Frame-rate: 8 frames per second. Also, see Supplementary Figure 7a.

File Name: Supplementary Movie 2

Description: U2OS-Lamp1-mCherry expressing cells were transfected with siRNA control and chased every minute after 15 minutes of complete amino acid starvation for up to 147 minutes, using time-lapse fluorescence microscopy. 10 μm scale bar is shown in the first frame. Numbers indicate the time (minutes) of amino acid starvation. Frame-rate: 8 frames per second.

File Name: Supplementary Movie 3

Description: U2OS-Lamp1-mCherry expressing cells were transfected with siRNA against Rap1A+B and chased every minute after 15 minutes of complete amino acid starvation for up to 239 minutes, using time-lapse fluorescence microscopy. 10 μm scale bar is shown in the first frame. Numbers indicate the time (minutes) of amino acid starvation. Frame-rate: 8 frames per second. Also, see Supplementary Figure 7a.

File Name: Supplementary Movie 4

Description: Time-lapse confocal video capture on a single plane of U2OS cells with LAMP1-mCherry marked lysosomes that had been transfected with control siRNA. 10 μm scale bar is shown in the first frame. Numbers indicate the time (seconds) from the first frame. Frame-rate: 8 frames per second. Image of cell is also shown in Supplementary Figure 7c.

File Name: Supplementary Movie 5

Description: Time-lapse confocal video capture on a single plane of U2OS cells with LAMP1-mCherry marked lysosomes that had been transfected with control siRNA and starved of all amino acids

for 6.5 hours. 10 m scale bar is shown in the first frame. Numbers indicate the time (seconds) from the first frame. Frame-rate: 8 frames per second. Image of cell is also shown in Supplementary Figure 7c.

File Name: Supplementary Movie 6

Description: Time-lapse confocal video capture on a single plane of U2OS cells with LAMP1-mCherry marked lysosomes that had been transfected with Rap1A+B siRNA and starved of all amino acids for 6.5 hours. 10 m scale bar is shown in the first frame. Numbers indicate the time (seconds) from the first frame. Frame-rate: 8 frames per second. Image of cell is also shown in Supplementary Figure 7c.

File Name: Supplementary Movie 7

Description: Time-lapse confocal video capture on a single plane of U2OS cells with LAMP1-mCherry marked lysosomes that had been treated with DMSO control for 30 minutes. 10 m scale bar is shown in the first frame. Numbers indicate the time (seconds) from the first frame. Framerate: 8 frames per second. Image of cell is also shown in Supplementary Figure 11a.

File Name: Supplementary Movie 8

Description: Time-lapse confocal video capture on a single plane of U2OS cells with LAMP1-mCherry marked lysosomes that had been treated with 0.1 M Lat A for 30 minutes. 10 m scale bar is shown in the first frame. Numbers indicate the time (seconds) from the first frame. Frame-rate: 8 frames per second. Image of cell is also shown in Supplementary Figure 11a.

File Name: Supplementary Movie 9

Description: Time-lapse confocal video capture on a single plane of U2OS cells with LAMP1-mCherry marked lysosomes expressing LifeAct-EGFP that had been treated with DMSO for 30 minutes. 1 m scale bar is shown in the first frame. White arrowheads in the first frame indicate lysosome-associated actin. Numbers indicate the time (seconds) from the first frame. Framerate: 8 frames per second. Image of cell is also shown in Supplementary Figure 11c.

File Name: Supplementary Movie 10

Description: Time-lapse confocal video capture on a single plane of U2OS cells with LAMP1-mCherry

marked lysosomes expressing LifeAct-EGFP that had been treated with 0.1 μM Lat A for 30 minutes. 1 μm scale bar is shown in the first frame. Numbers indicate the time (seconds) from the first frame. Frame-rate: 8 frames per second. Image of cell is also shown in Supplementary Figure 11c.

File Name: Supplementary Movie 11

Description: Time-lapse confocal video capture on a single plane of control siRNA transfected HEK293A cells expressing LifeAct-EGFP. 1 μm scale bar is shown in the first frame. Numbers indicate the time (seconds) from the first frame. Frame-rate: 8 frames per second. Image of cell is also shown in Supplementary Figure 11d.

File Name: Supplementary Movie 12

Description: Time-lapse confocal video capture on a single plane of siRNA Rap1A+B depleted HEK293A cells expressing LifeAct-EGFP. 1 μm scale bar is shown in the first frame. Numbers indicate the time (seconds) from the first frame. Frame-rate: 8 frames per second. Image of cell is also shown in Supplementary Figure 11d.

File Name: Supplementary Movie 13

Description: Time-lapse confocal video capture on a single plane of U2OS cells with LAMP1-mCherry marked lysosomes that had been transfected with control siRNA. 10 μm scale bar is shown in the first frame. Numbers indicate the time (seconds) from the first frame. Frame-rate: 8 frames per second. Image of cell is also shown in Supplementary Figure 11f.

File Name: Supplementary Movie 14

Description: Time-lapse confocal video capture on a single plane of U2OS cells with LAMP1-mCherry marked lysosomes that had been transfected with Rap1A+B siRNA. 10 μm scale bar is shown in the first frame. Numbers indicate the time (seconds) from the first frame. Frame-rate: 8 frames per second. Image of cell is also shown in Supplementary Figure 11f.

File Name: Supplementary Movie 15

Description: U2OS cells were transfected with Lifeact to visualize F-actin (1) and chased every minute after 10 minutes of complete amino acid starvation for up to 190 minutes, using time-laps

fluorescence microscopy. 10 μm scale bar is shown in the first frame. Numbers indicate the time (minutes) of amino acid starvation. Frame-rate: 8 frames per second.

File Name: Supplementary Movie 16

Description: Time-laps video of U2OS cells with LAMP1-mCherry marked lysosomes that were amino acid starved for 3.5h and treated with 0.1 μM latrunculin A (Lat A) for an additional 30 minutes (total 4 hours of starvation). Time-lap video starts from 150 minutes of amino acid starvation. Numbers indicate the time of amino acid starvation (-AA, minutes) and LatA treatment (LatA, minutes). 10 μm scale bar is shown in the first frame. Frame-rate: 8 frames per second.
