# Supplementary material for: Rap1-GTPases control mTORC1 activity by coordinating lysosome organization with amino acid availability
Source: Nat Commun. 2020 Mar 17;11:1416. doi: 10.1038/s41467-020-15156-5 (PMC7078236; doi:10.1038/s41467-020-15156-5)
Supplement: Supplementary file 19 — Reporting Summary [file 41467_2020_15156_MOESM19_ESM.pdf]

nature research

Corresponding author(s): Anders Mutvei, John Blenis

Reporting Summary

Nature Research wishes to improve the reproducibility of the work that we publish. This form provides structure for consistency and transparency in reporting. For further information on Nature Research policies, see Authors & Referees and the Editorial Policy Checklist.

Statistical parameters

When statistical analyses are reported, confirm that the following items are present in the relevant location (e.g. figure legend, table legend, main text, or Methods section).

n/a	Confirmed	
☐	☒	The exact sample size (*n*) for each experimental group/condition, given as a discrete number and unit of measurement
☐	☒	An indication of whether measurements were taken from distinct samples or whether the same sample was measured repeatedly
☐	☒	The statistical test(s) used AND whether they are one- or two-sided<br>*Only common tests should be described solely by name; describe more complex techniques in the Methods section.*
☒	☐	A description of all covariates tested
☒	☐	A description of any assumptions or corrections, such as tests of normality and adjustment for multiple comparisons
☐	☒	A full description of the statistics including central tendency (e.g. means) or other basic estimates (e.g. regression coefficient) AND variation (e.g. standard deviation) or associated estimates of uncertainty (e.g. confidence intervals)
☒	☐	For null hypothesis testing, the test statistic (e.g. *F*, *t*, *r*) with confidence intervals, effect sizes, degrees of freedom and *P* value noted<br>*Give P values as exact values whenever suitable.*
☒	☐	For Bayesian analysis, information on the choice of priors and Markov chain Monte Carlo settings
☒	☐	For hierarchical and complex designs, identification of the appropriate level for tests and full reporting of outcomes
☒	☐	Estimates of effect sizes (e.g. Cohen's *d*, Pearson's *r*), indicating how they were calculated
☐	☒	Clearly defined error bars<br>*State explicitly what error bars represent (e.g. SD, SE, CI)*

Our web collection on statistics for biologists may be useful.

Software and code

Policy information about availability of computer code

Data collection

Immunoblot images were captured using an Odyssey CLx Infrared Imaging System and the software Image Studio (v1.0.11,LI-COR Biosciences) v1.0.11), except for in Fig S1b, where images were captured with an Amersham Imager 680 (GE Healthcare), using SuperSignal West Pico PLUS Chemiluminescent Substrate from Thermo Scientific.

Confocal images were captured with a Zeiss LSM 880 Laser Scanning Confocal Microscope and the ZEN software (Zeiss). Live-images were collected on a Nikon Eclipse Ti microscope equipped with an Andor Zyla sCMOS camera, using the 60x oil immersion objective and NIS-Elements AR software (Nikon)

qPCR analysis was carried out on a QuantStudio6 Flex and the QuantStudio Real-Time PCR SOftware (v.1.0)

Data analysis

The endosomal distribution was quantified in an automated way using ImageJ/Fiji (v1.51e).
Average pixel intensity was determined in Fiji/ImageJ (v1.51e).
Vesicle trafficking was analyzed using TrackMate (v3.4.0) for Fiji/ImageJ
3D-reconstructions were created in Imaris (Bitplane, v. x64 8.3.1).
Statisitical tests were carried out in R-studio (Version 0.99.896), Microsoft Excel 2016 or GraphPad Prism (Version 8.0.0, 224).
Data were plotted in Microsoft Excel 2016 MSO (16.0.4405.1000) 64-bit and GraphPad Prism (Version 8.0.0, 224)

For manuscripts utilizing custom algorithms or software that are central to the research but not yet described in published literature, software must be made available to editors/reviewers upon request. We strongly encourage code deposition in a community repository (e.g. GitHub). See the Nature Research guidelines for submitting code & software for further information.

Data

Policy information about availability of data

All manuscripts must include a data availability statement. This statement should provide the following information, where applicable:
- Accession codes, unique identifiers, or web links for publicly available datasets
- A list of figures that have associated raw data
- A description of any restrictions on data availability

The data that support the findings of this study are available from the corresponding author upon reasonable request.

Field-specific reporting

Please select the best fit for your research. If you are not sure, read the appropriate sections before making your selection.

[x] Life sciences [ ] Behavioural & social sciences

For a reference copy of the document with all sections, see nature.com/authors/policies/ReportingSummary-flat.pdf

Life sciences

Study design

All studies must disclose on these points even when the disclosure is negative.

Sample size	Sample sizes were not pre-calucalated but are comparable to similar studies in the field. Sample size and number of independent experiments are stated in the figure captions or in the methods section and Source Data file. Statistical analyses were only performed on experiments repeated at least three times except for in electron microscopy analyses, where individual lysosomes or cells were analyzed across two independent experiments.
Data exclusions	No data was excluded for analysis.
Replication	The replication number is indicated in the legend of the corresponding figure. All experiments were replicated successfully with similar results.
Randomization	No methods of randomization were used.
Blinding	Investigators were not blinded to group allocation during data collection and analysis, except for in Fig 4c-d and Sup Fig 11d-e, where the scorer was blinded to treatment conditions represented in the images.

Materials & experimental systems

Policy information about availability of materials

n/a	Involved in the study	
[x]	[ ]	Unique materials
[ ]	[x]	Antibodies
[ ]	[x]	Eukaryotic cell lines
[x]	[ ]	Research animals
[x]	[ ]	Human research participants

Unique materials

Obtaining unique materials	*Describe any restrictions on the availability of unique materials OR confirm that all unique materials used are readily available from the authors or from standard commercial sources (and specify these sources).*

Antibodies

Antibodies used	Western Blot<br>The following antibodies were purchased from Cell Signaling Technologies and used in a 1:1000 dilution: phospho p70 S6K1Thr389 (9234), S6K1 (2708), phospho 4E-BP1 Ser65 (9451), mTOR (2983), phospho Akt Ser473 (4060), Akt1 (2967), COX IV (4850), phospho PRAS40 Thr246 (2997), Rap1A+B (2399), Rap1B (2326), phospho AMPKalpha Thr172 (2535), LAMP1 (9091), AMPKalpha (2603), RagA (4357), RagC (3360), RagD (4470), Raptor (2280), p14/LAMTOR2/ROBLD3 (8145). Additional antibodies used in 1:1000 dilution: phospho GCN2 Thr899 (Abcam: ab75836), GCN2 (Novus Biologicals: MAB6878), LAMP2 (Santa Cruz

Biotechnology: sc-18822), TFEB (Bethyl Laboratories, A303-673A), HA-probe (Santa Cruz Biotechnology: sc-805), LC3B (Novus Biologicals: NB100-2220), Calnexin (Abcam: ab75801), B-act (Abcam: ab8226). Antibodies used in 1:5000 dilutions: Anti-FLAG M2 (Sigma-Aldrich: F3165), GAPDH (Sigma-Aldrich: G8795).

In Supplementary Figure 1b, the following horseradish peroxidase linked secondary antibodies were purchased from GE Healthcare and used in 1:5000 dilutions: Anti-Mouse igG (NXA931V) and Anti-Rabbit IgG (NA934V). For all other western blots, secondary antibodies were purchased from LI-COR Biosciences and used in 1:10000 dilutions: Donkey Anti-Mouse IgG IRDye 680LT (926-68022), Donkey Anti-Rabbit IgG Antibody IRDye 800CW (926-32213).

Immunofluorescence:
The following antibodies were used in 1:100 dilutions (validation refers to demonstration that immunolabeling is lost with siRNA knockdown or knockout of the antigen of interest): LAMP1 (Cell Signaling Technologies, 9091), LAMP2 (Santa Cruz Biotechnology, H4B4 sc-18822), mTOR (Cell Signaling Technologies, 2983), CD63 (H5C6, BD Bioscience), RagC (Cell Signaling Technologies, 3360), DYKDDDDK Tag (Cell Signaling Technologies ,14793), Rab7 (Cell Signaling Technologies, 9367), a-tubulin (Sigma-Aldrich: T6199), Rab5 (Cell Signaling Technologies, 3547). LC3B (Cell Signaling Technologies, 3868), Anti-FLAG M2 (Sigma-Aldrich: F3165) was used in a 1:800 dilution and DAPI (Biotium) at a final concentration of 1 ug/mL.

Validation

All antibodies used were bought from commercial companies and are widely used for similar experiments by other researchers worldwide. We have also validated the following antibodies in this study (validation refers to demonstration that immunolabeling is lost with siRNA knockdown or knockout of the antigen of interest): Rap1A+B (CST2399), Rap1B (CST2326) LAMP1 (Cell Signaling Technologies, 9091), LAMP2 (Santa Cruz Biotechnology, H4B4 sc-18822), mTOR (CST, 2983) and Rab7 (Cell Signaling Technologies, 9367) (Fig1, Supplementary Figure 13-14).
Original links to each antibody are listed below:
https://www.cellsignal.com/products/primary-antibodies/p70-s6-kinase-49d7-rabbit-mab/2708
https://www.cellsignal.com/products/primary-antibodies/phospho-p70-s6-kinase-thr389-108d2-rabbit-mab/9234
https://www.cellsignal.com/products/primary-antibodies/phospho-4e-bp1-ser65-antibody/9451
https://www.cellsignal.com/products/primary-antibodies/phospho-akt-ser473-d9e-xp-rabbit-mab/4060
https://www.cellsignal.com/products/primary-antibodies/akt1-2h10-mouse-mab/2967
https://www.cellsignal.com/products/primary-antibodies/cox-iv-3e11-rabbit-mab/4850
https://www.cellsignal.com/products/primary-antibodies/phospho-pras40-thr246-c77d7-rabbit-mab/2997
https://www.cellsignal.com/products/primary-antibodies/phospho-ampka-thr172-40h9-rabbit-mab/2535
https://www.cellsignal.com/products/primary-antibodies/ampka-23a3-rabbit-mab/2603
https://www.cellsignal.com/products/primary-antibodies/raga-d8b5-rabbit-mab/4357
https://www.cellsignal.com/products/primary-antibodies/ragc-antibody/3360
https://ocsuat-en.cellsignal.jp/products/primary-antibodies/ragd-antibody/4470
https://www.cellsignal.com/products/primary-antibodies/raptor-24c12-rabbit-mab/2280
https://www.cellsignal.com/products/primary-antibodies/lamtor2-robld3-d7c10-rabbit-mab/8145
https://www.abcam.com/gcn2-phospho-t899-antibody-epr2320y-ab75836.html
https://www.novusbio.com/products/gcn2-antibody-708210_mab6878
https://www.bethyl.com/product/A303-673A/TFEB+Antibody
https://www.scbt.com/p/ha-probe-antibody-y-11
https://www.novusbio.com/products/lc3b-antibody_nb100-2220
https://www.abcam.com/calnexin-antibody-er-membrane-marker-ab75801.html
https://www.abcam.com/beta-actin-antibody-mabcam-8226-loading-control-ab8226.html
https://www.sigmaaldrich.com/catalog/product/sigma/f3165
https://www.sigmaaldrich.com/catalog/product/sigma/g8795
https://www.bdbiosciences.com/us/reagents/research/antibodies-buffers/immunology-reagents/anti-human-antibodies/cell-surface-antigens/purified-mouse-anti-human-cd63-h5c6/p/556019
https://www.cellsignal.com/products/primary-antibodies/dykddddk-tag-d6w5b-rabbit-mab-binds-to-same-epitope-as-sigma-s-
https://www.sigmaaldrich.com/catalog/product/sigma/t6199
https://www.cellsignal.com/products/primary-antibodies/rab5-c8b1-rabbit-mab/3547
https://www.cellsignal.com/products/primary-antibodies/lc3b-d11-xp-rabbit-mab/3868

Eukaryotic cell lines

Policy information about cell lines

Cell line source(s)

HELA, HEK293T, U2OS, mouse embryonic fibroblasts (MEF) cells deficient in GCN2 and their littermate-derived wild-type control cells were obtained from ATCC.
HEK293A cells were purchased from Thermo Fisher Scientific.
RagA/B CRISPR/Cas9 knockout cells and control cells were kindly provided by Drs. Kun-Liang Guan and Jenna L. Jewell (University of California San Diego).
Mouse embryonic fibroblasts deficient in p14/LAMTOR2 and wild-type control cells were kindly provided by Drs. David Sabatini (Whitehead Institute) and Brendan Manning (Harvard School of Public Health).
U2OS cells stably expressing mCherry-LAMP1 were generated through transfection of pLAMP1-mCherry and selection.

Authentication	Commercially available cell lines were provided with authenticity forms from the provider. No additional authentication was carried out. Cell lines generated by other labs were validated by immunoblotting for the protein of interest that had been knocked out.
Mycoplasma contamination	Cell lines were routinely checked for mycoplasma and were always found to be negative.
Commonly misidentified lines (See ICLAC register)	To our knowledge, no cell lines used in this study are listed in the database of commonly misidentified cell lines (ICLAC).

Research animals

Policy information about studies involving animals; ARRIVE guidelines recommended for reporting animal research

Animals/animal-derived materials	*For laboratory animals, report species, strain, sex and age OR for animals observed in or captured from the field, report species, sex and age where possible.*

Human research participants

Policy information about studies involving human research participants

Population characteristics	*Describe the covariate-relevant population characteristics of the human research participants (e.g. age, gender, genotypic information, past and current diagnosis and treatment categories).*

Method-specific reporting

n/a	Involved in the study
☒	☐ ChIP-seq
☒	☐ Flow cytometry
☒	☐ Magnetic resonance imaging

ChIP-seq

Data deposition

☐ Confirm that both raw and final processed data have been deposited in a public database such as GEO.

☐ Confirm that you have deposited or provided access to graph files (e.g. BED files) for the called peaks.

Data access links *May remain private before publication.*	*For "Initial submission" or "Revised version" documents, provide reviewer access links. For your "Final submission" document, provide a link to the deposited data.*
Files in database submission	*Provide a list of all files available in the database submission.*
Genome browser session (e.g. UCSC)	*Provide a link to an anonymized genome browser session for "Initial submission" and "Revised version" documents only, to enable peer review. Write "no longer applicable" for "Final submission" documents.*

Methodology

Replicates	*Describe the experimental replicates, specifying number, type and replicate agreement.*
Sequencing depth	*Describe the sequencing depth for each experiment, providing the total number of reads, uniquely mapped reads, length of reads and whether they were paired- or single-end.*
Antibodies	*Describe the antibodies used for the ChIP-seq experiments; as applicable, provide supplier name, catalog number, clone name, and lot number.*
Peak calling parameters	*Specify the command line program and parameters used for read mapping and peak calling, including the ChIP, control and index files used.*
Data quality	*Describe the methods used to ensure data quality in full detail, including how many peaks are at FDR 5% and above 5-fold enrichment.*
Software	*Describe the software used to collect and analyze the ChIP-seq data. For custom code that has been deposited into a community repository, provide accession details.*

Flow Cytometry

Plots

Confirm that:

☐ The axis labels state the marker and fluorochrome used (e.g. CD4-FITC).

- [ ] The axis scales are clearly visible. Include numbers along axes only for bottom left plot of group (a 'group' is an analysis of identical markers).
- [ ] All plots are contour plots with outliers or pseudocolor plots.
- [ ] A numerical value for number of cells or percentage (with statistics) is provided.

Methodology

Sample preparation	*Describe the sample preparation, detailing the biological source of the cells and any tissue processing steps used.*
Instrument	*Identify the instrument used for data collection, specifying make and model number.*
Software	*Describe the software used to collect and analyze the flow cytometry data. For custom code that has been deposited into a community repository, provide accession details.*
Cell population abundance	*Describe the abundance of the relevant cell populations within post-sort fractions, providing details on the purity of the samples and how it was determined.*
Gating strategy	*Describe the gating strategy used for all relevant experiments, specifying the preliminary FSC/SSC gates of the starting cell population, indicating where boundaries between "positive" and "negative" staining cell populations are defined.*

- [ ] Tick this box to confirm that a figure exemplifying the gating strategy is provided in the Supplementary Information.

Magnetic resonance imaging

Experimental design

Design type	*Indicate task or resting state; event-related or block design.*
Design specifications	*Specify the number of blocks, trials or experimental units per session and/or subject, and specify the length of each trial or block (if trials are blocked) and interval between trials.*
Behavioral performance measures	*State number and/or type of variables recorded (e.g. correct button press, response time) and what statistics were used to establish that the subjects were performing the task as expected (e.g. mean, range, and/or standard deviation across subjects).*

Acquisition

Imaging type(s)	*Specify: functional, structural, diffusion, perfusion.*
Field strength	*Specify in Tesla*
Sequence & imaging parameters	*Specify the pulse sequence type (gradient echo, spin echo, etc.), imaging type (EPI, spiral, etc.), field of view, matrix size, slice thickness, orientation and TE/TR/flip angle.*
Area of acquisition	*State whether a whole brain scan was used OR define the area of acquisition, describing how the region was determined.*

Diffusion MRI ☐ Used ☐ Not used

Preprocessing

Preprocessing software	*Provide detail on software version and revision number and on specific parameters (model/functions, brain extraction, segmentation, smoothing kernel size, etc.).*
Normalization	*If data were normalized/standardized, describe the approach(es): specify linear or non-linear and define image types used for transformation OR indicate that data were not normalized and explain rationale for lack of normalization.*
Normalization template	*Describe the template used for normalization/transformation, specifying subject space or group standardized space (e.g. original Talairach, MNI305, ICBM152) OR indicate that the data were not normalized.*
Noise and artifact removal	*Describe your procedure(s) for artifact and structured noise removal, specifying motion parameters, tissue signals and physiological signals (heart rate, respiration).*
Volume censoring	*Define your software and/or method and criteria for volume censoring, and state the extent of such censoring.*

Statistical modeling & inference

Model type and settings	*Specify type (mass univariate, multivariate, RSA, predictive, etc.) and describe essential details of the model at the first and second levels (e.g. fixed, random or mixed effects; drift or auto-correlation).*
Effect(s) tested	*Define precise effect in terms of the task or stimulus conditions instead of psychological concepts and indicate whether ANOVA or factorial designs were used.*

Specify type of analysis: ☐ Whole brain ☐ ROI-based ☐ Both

Statistic type for inference
(See Eklund et al. 2016)

Specify voxel-wise or cluster-wise and report all relevant parameters for cluster-wise methods.

Correction

Describe the type of correction and how it is obtained for multiple comparisons (e.g. FWE, FDR, permutation or Monte Carlo).

Models & analysis

n/a	Involved in the study
☐	☐ Functional and/or effective connectivity
☐	☐ Graph analysis
☐	☐ Multivariate modeling or predictive analysis

Functional and/or effective connectivity

Report the measures of dependence used and the model details (e.g. Pearson correlation, partial correlation, mutual information).

Graph analysis

Report the dependent variable and connectivity measure, specifying weighted graph or binarized graph, subject- or group-level, and the global and/or node summaries used (e.g. clustering coefficient, efficiency, etc.).

Multivariate modeling and predictive analysis

Specify independent variables, features extraction and dimension reduction, model, training and evaluation metrics.

Behavioural & social sciences

Study design

All studies must disclose on these points even when the disclosure is negative.

Study description

Briefly describe the study type including whether data are quantitative, qualitative, or mixed-methods (e.g. qualitative cross-sectional, quantitative experimental, mixed-methods case study).

Research sample

State the research sample (e.g. Harvard university undergraduates, villagers in rural India) and provide relevant demographic information (e.g. age, sex) and indicate whether the sample is representative. Provide a rationale for the study sample chosen. For studies involving existing datasets, please describe the dataset and source.

Sampling strategy

Describe the sampling procedure (e.g. random, snowball, stratified, convenience). Describe the statistical methods that were used to predetermine sample size OR if no sample-size calculation was performed, describe how sample sizes were chosen and provide a rationale for why these sample sizes are sufficient. For qualitative data, please indicate whether data saturation was considered, and what criteria were used to decide that no further sampling was needed.

Data collection

Provide details about the data collection procedure, including the instruments or devices used to record the data (e.g. pen and paper, computer, eye tracker, video or audio equipment) whether anyone was present besides the participant(s) and the researcher, and whether the researcher was blind to experimental condition and/or the study hypothesis during data collection.

Timing

Indicate the start and stop dates of data collection. If there is a gap between collection periods, state the dates for each sample cohort.

Data exclusions

If no data were excluded from the analyses, state so OR if data were excluded, provide the exact number of exclusions and the rationale behind them, indicating whether exclusion criteria were pre-established.

Non-participation

State how many participants dropped out/declined participation and the reason(s) given OR provide response rate OR state that no participants dropped out/declined participation.

Randomization

If participants were not allocated into experimental groups, state so OR describe how participants were allocated to groups, and if allocation was not random, describe how covariates were controlled.
